# The triple-deck stage of marginal separation

**DOI:** 10.1007/s10665-021-10125-3

**Published:** 2021-06-01

**Authors:** Stefan Braun, Stefan Scheichl, Dominik Kuzdas

**Affiliations:** 1 TU Wien, Institute of Fluid Mechanics and Heat Transfer, Getreidemarkt 9, 1060 Vienna, Austria; 2 Magna Powertrain, Wienersdorfer Straße 20–24, 2514 Traiskirchen, Austria

**Keywords:** Chebyshev collocation method, Finite-time blow-up, Interaction boundary layer theory, Laminar separation bubble, Laminar–turbulent transition, Unsteady separation

## Abstract

The method of matched asymptotic expansions is applied to the investigation of transitional separation bubbles. The problem-specific Reynolds number is assumed to be large and acts as the primary perturbation parameter. Four subsequent stages can be identified as playing key roles in the characterization of the incipient laminar–turbulent transition process: due to the action of an adverse pressure gradient, a classical laminar boundary layer is forced to separate marginally (I). Taking into account viscous–inviscid interaction then enables the description of localized, predominantly steady, reverse flow regions (II). However, certain conditions (e.g. imposed perturbations) may lead to a finite-time breakdown of the underlying reduced set of equations. The ensuing consideration of even shorter spatio-temporal scales results in the flow being governed by another triple-deck interaction. This model is capable of both resolving the finite-time singularity and reproducing the spike formation (III) that, as known from experimental observations and direct numerical simulations, sets in prior to vortex shedding at the rear of the bubble. Usually, the triple-deck stage again terminates in the form of a finite-time blow-up. The study of this event gives rise to a noninteracting Euler–Prandtl stage (IV) associated with unsteady separation, where the vortex wind-up and shedding process takes place. The focus of the present paper lies on the triple-deck stage III and is twofold: firstly, a comprehensive numerical investigation based on a Chebyshev collocation method is presented. Secondly, a composite asymptotic model for the regularization of the ill-posed Cauchy problem is developed.

**Supplementary Information:**

The online version contains supplementary material available at 10.1007/s10665-021-10125-3.

## Introduction

The present paper is concerned with the investigation of the early stages of laminar–turbulent transition induced by localized boundary layer separation. Notably in this regard, the analysis will be approached from the perspective of high Reynolds number (${\text{ Re }}$) asymptotics. The mentioned type of transition may be observed e.g. in the flow past the leading-edge suction side of an airfoil. Here, the angle of attack $\alpha$ is kept almost fixed at a relatively small value (or may change in a quasi-steady fashion) and the main, predominantly inviscid, part of the flow remains steady and follows the streamlined body contour. This in various practical applications quite common setting is distinct from the more complex ‘dynamical stall’ flow phenomenon that manifests itself on an airfoil during rapid, transient motion in which the angle of attack exceeds the static stall limit value [1, 2]. Whereas the latter flow situation is characterized by the formation of large dynamic stall vortices of size comparable with the chord, associated massive alterations of the aerodynamic loads (e.g. augmented lift) and stall hysteresis effects, vortical structures in the present context are small-scaled, remain confined to the thin shear layer adjacent to the body surface and lift is affected insignificantly only.

There are many possible routes to turbulence. In the present context, one may roughly distinguish between two: In the first, ‘natural’, scenario, initially two-dimensional instabilities, the so-called Tollmien–Schlichting (TS) waves, develop in a *fully attached* laminar boundary layer due to the presence of external disturbances (e.g. sound waves, surface roughnesses, structure vibrations, freestream turbulence, etc.). The downstream travelling TS waves gradually grow in amplitude until they break down to form three-dimensional vortical structures and, eventually, turbulent spots appear. Since this transition mechanism typically is triggered by small perturbations, at least the initial stage can be described by linear stability theory based on the parallel flow assumption, which is well established in the literature, see e.g. [3]. A rigorous high ${\text{ Re }}$ asymptotic analysis of the TS wave generation process, the so-called *receptivity problem*, leads to the conventional triple-deck scalings originally discovered by Stewartson [4], Stewartson and Williams [5], Neiland [6] and Messiter [7] in completely different contexts, see Goldstein [8, 9] and Ruban [10]. However, a thorough theoretical understanding of the evolution towards full transition via this route is still lacking.

In contrast to this seemingly ‘slowly’ evolving scenario, the second route, i.e. the one we focus on, starts from a laminar boundary layer, but *on the verge of separation*. Whereas separation and turbulence are two different things, it turns out that separation very greatly enhances the propensity towards the formation of turbulence. Due to the presence of an internal inflection point in the velocity profile of the separated shear layer, the flow is highly unstable from the very beginning and inherently requires a nonlinear description to fully resolve the underlying physical mechanisms. Above all, this pathway to turbulence bypasses the development of TS waves (therefore may be called ‘bypass transition’) and runs through a cascade of characteristic stages with distinct length and time scales which become progressively shorter [11].

The increased use of high-tenacity materials like carbon-fibre-reinforced composites allows the realization of relatively thin airfoils with the objective to minimize viscous drag. Their lift coefficient is proportional to the angle of attack $\alpha$ for $\alpha \ll 1$. However, they are prone to the occurrence of a laminar separation bubble (LSB) at their leading-edge suction side when $\alpha$ exceeds a certain value which still is small. Since LSBs may trigger rapid transition under certain conditions, their development crucially impacts the drag because the transition location then ‘jumps’ from its usual, ‘natural’ position at the mid to rear section to almost the leading edge of the airfoil. A further increase of $\alpha$ eventually leads to the entire disintegration of the LSB with the consequence of abrupt and complete loss of lift (stall) and a substantial gain of drag. These circumstances explain the undiminished research interest in this phenomenon over more than eighty-five years, cf. the paper of Jones [12], who was the first to discover the phenomenon of shallow separation bubbles on airfoils experimentally in a wind tunnel facility.

**Fig. 1:**
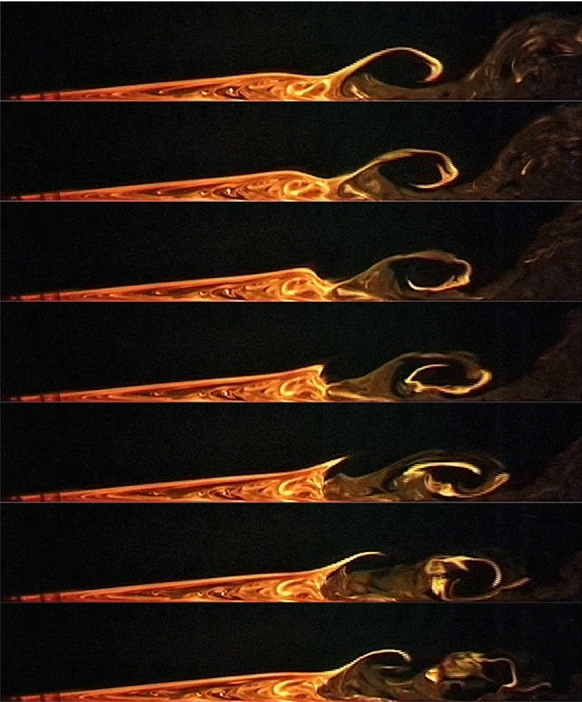
Colour visualization of a transitional separation bubble at the bottom wall of a laminar water tunnel: successive snapshots during a full $\varLambda$-vortex generation/disintegration cycle (side view, flow from left to right, characteristic Reynolds number ${\text{ Re }}\approx 10^5$). *Source* U. Rist & M. Lang, Institute of Aerodynamics and Gas Dynamics, University of Stuttgart

As is well known, in practice high ${\text{ Re }}$ flows are turbulent, and hence one can expect that there will be a natural tendency for structures with small length and short time scales to develop. Scientific observations indicate the formation of well-defined, coherent vortical structures, the so-called $\varLambda$-vortices or ‘rollers’, in the rear region of the bubble that are convected downstream, see e.g. [13–15], and Fig. 1. Therefore, it is obvious that there is an inherent unsteady behaviour related to LSBs.

The mentioned structures, of size comparable with the laminar boundary layer thickness, convey the early stage of the turbulent part of the flow and persist a certain distance downstream of the mean reattachment point until they disintegrate into small-scale vortices (the ‘cloudy’ pattern at the top right of Fig. 1) characteristic of the developing turbulence. Again, this laminar–turbulent transition scenario generated by a locally detached shear layer is not yet fully understood, but it is commonly accepted that the repetitive bursting of a LSB, i.e. the (largely) periodic vortex shedding at its rear has to play a key role insofar as it precedes and initiates rather than follows full transition, see e.g. [16].

The genesis of a rigorous high ${\text{ Re }}$ asymptotic framework for the description of (steady, planar) laminar separation bubbles dates back to the early 1980s in the course of the vibrant development of triple-deck theory and its application to fundamental flow problems in those days [17–19]. Considerable efforts were undertaken to extend the promising approaches with regard to time dependence [20, 21], and the third spatial dimension (spanwise direction), e.g. [22–25]. One of the great challenges in dealing with high-${\text{ Re }}$ flows is their susceptibility not only to separation but also to instability. Specifically, the nonlinear evolution equations which arise from the asymptotic analysis typically exhibit stiff behaviour and, even when starting from perfectly smooth initial data, their solutions may eventually develop singularities [26]. In the present context, the progress was hampered by the occurrence of difficulties in the sense that the simplified mathematical models resulting from the perturbation approach turned out to be ‘ill-posed’ [27]. Regularization methods in this regard were not yet known and moreover the computing power available at that time was still insufficient to solve the occasionally delicate nonlinear evolution equations numerically. Of course, high-end computing power is available now, and as far as the ill-posedness is concerned, we believe these difficulties can be overcome as well [28].

**Fig. 2:**
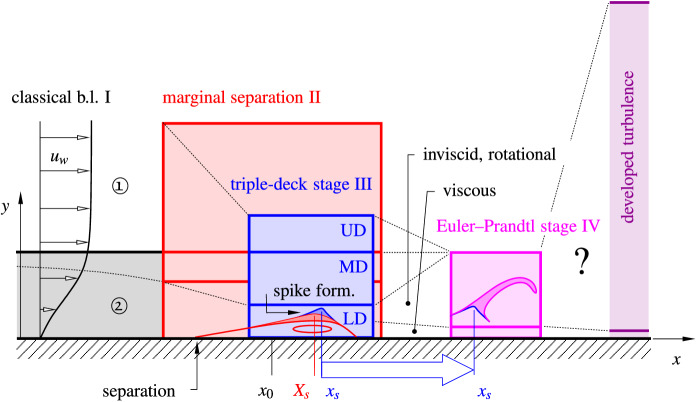
High Reynolds number asymptotic (layer) structure of incipient laminar–turbulent transition in laminar separation bubbles (schematic, cf. Fig. 1). Classical laminar boundary layer at the verge of separation (black): inviscid outer flow $\bigcirc \mathfrak {1}$, viscous region $\bigcirc \mathfrak {2}$. Consecutive interactive stages of separation (red) and spike formation (blue), followed by self-induced vortex wind-up (magenta, shifted to the right for better illustration) accompanied by finite-time blow-up events, here modelled up to the Euler–Prandtl stage. Long-term objective: linkage to the time-mean two-tiered turbulent boundary layer flow description [30, 31], (purple)

From the current asymptotic viewpoint, the bursting process of separation bubbles evolves through a sequence of stages, Fig. 2. Each of them can be posed as a singular perturbation problem to be solved by application of matched asymptotic expansions. The involved spatial and temporal scales relevant in each stage and, consequently, the dominating physical mechanisms appear in a natural way. Most important, the perturbation approach leads to *universally valid* results in the form of similarity laws independent of the specific flow under consideration, which, nevertheless, require numerical treatment. As shown below in more detail, the successive passage of these distinct stages clearly exhibits features of a developing turbulent boundary layer: the shift of viscous effects to a wall layer of decreasing thickness and the release of vortices into a detached, predominantly inviscid, rotational region of increasing thickness. During these early stages of the transition process the overall flow structure, comprising an outer inviscid flow field and a viscous boundary layer adjacent to the solid wall, is retained and not destroyed by bubble bursting (as for e.g. in the case of massive, break-away separation, not discussed here).

Section 2 serves to present essential preliminary work regarding the high ${\text{ Re }}$ asymptotic description of LSBs in the perspective of the present approach and to motivate the starting point of our study. Readers who are familiar with marginal separation theory can skip this chapter and start with page 8 or 9. Section 3 examines and extends the pivotal work of Elliott and Smith [29] as related to a systematic numerical investigation of the spike formation stage and its finite-time breakdown. Moreover, a composite asymptotic model is presented in Sect. 4 for the purpose of resolving the ill-posed Cauchy problem associated with the current triple-deck formulation. The noninteractive Euler–Prandtl stage which is initiated by the finite-time blow-up of the preceding triple-deck stage is outlined in Sect. 5. The main findings of the present work are briefly summarized and discussed in Sect. 6 and more detailed descriptions of the asymptotic layer structure of the triple-deck stage and the applied numerical technique are provided in Appendices A and B, respectively.

## From classical to interaction boundary layer theory

Here we briefly outline the current status of how the onset of laminar–turbulent transition associated with localized boundary layer separation can be described by an asymptotic analysis. To this end the most important scalings, similarity laws (respective leading-order evolution equations), and main characteristics of each subsequent stage are summarized and discussed. The fundamental perturbation parameter of the present study is the Reynolds number

1 $$\begin{aligned} {\text{ Re }}=\frac{{\tilde{u}}_\infty {\tilde{L}}}{{\tilde{\nu }}}\gg 1, \end{aligned}$$

which is assumed to be large. More precisely, we consider the singular limit problem ${\text{ Re }}\rightarrow \infty$ of the Navier–Stokes equations applied to incipient separation and, for the sake of clarity and simplicity, restrict the treatment to planar, incompressible flows. Extensions of the theory to incorporate (quasi-) three-dimensional flow cases may be found in [22, 25, 32, 33]. The inclusion of compressibility effects is treated in [19, 34, 35] for strictly sub- and/or supersonic flows and in [36] for transonic flow conditions. Here and below the superscript tilde indicates dimensional quantities and ${\tilde{u}}_\infty$, ${\tilde{\nu }}$ and ${\tilde{L}}$ denote the freestream velocity, the kinematic viscosity of the fluid and a suitable reference length. The computational results are most conveniently expressed in terms of the elementary boundary layer characteristics displacement thickness $\delta ^*$ and wall shear stress $\tau _\mathrm{w}$,

2 $$\begin{aligned} \delta ^*={\text{ Re }}^{1/2}\,\frac{{\tilde{\delta }}^*}{{\tilde{L}}} = \int \limits _0^\infty \left( 1-\frac{U}{u_\mathrm{w}}\right) \,\mathrm{d}{\bar{y}},\quad \tau _\mathrm{w}={\text{ Re }}^{1/2}\frac{{\tilde{\tau }}_\mathrm{w}}{{\tilde{\rho }}{\tilde{u}}_\infty ^2}=\frac{\partial U}{\partial {\bar{y}}}\bigg |_{{\bar{y}}=0}, \end{aligned}$$

where $x={\tilde{x}}/{\tilde{L}}$, ${\bar{y}}={\text{ Re }}^{1/2}{\tilde{y}}/{\tilde{L}}$, ${\tilde{\rho }}=\text {const}$, $u_\mathrm{w}(x)$ and $U(x,{\bar{y}})$ denote the arc length measured from the leading edge, the usual boundary layer wall-normal coordinate, the density, the prescribed inviscid wall velocity and the boundary layer velocity profile in streamwise direction which satisfies the matching condition $U\rightarrow u_\mathrm{w}$ as ${\bar{y}}\rightarrow \infty$.

### Prandtl’s steady boundary layer at the verge of separation *(stage I)*

**Fig. 3:**
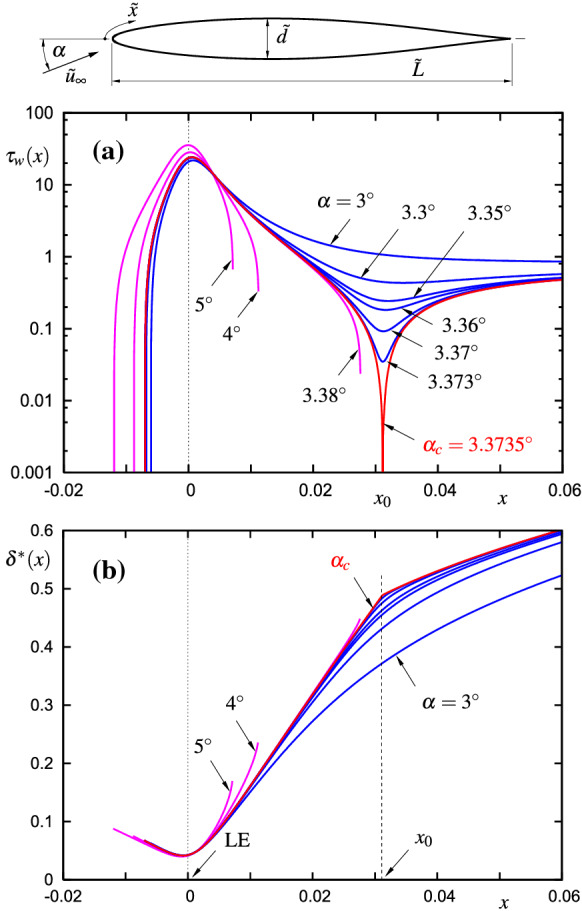
Steady classical boundary layer flow at the leading edge LE (suction side) of a specific symmetric airfoil with $d={\tilde{d}}/{\tilde{L}}=0.1$ relative thickness (airfoil data can be found in Supplementary online materials) in dependence of the angle of attack $\alpha$: **a** wall shear stress $\tau _\mathrm{w}$, **b** displacement thickness $\delta ^*$ according to (2) downstream of the front stagnation point

A comprehensive presentation of the subsequent stages of the early transition process necessarily includes the solution of Prandtl’s classical boundary layer equations as a starting point. To this end, the practically important example of the steady flow past the suction side of an airfoil at various small angles of attack $\alpha$ is considered, Fig. 3. As can be seen from Fig. 3a, an increase of $\alpha$ causes the wall shear distribution, indicated in blue, to form a local minimum in a region of adverse pressure gradient conditions (at about $3\%$ of the chord of the examined airfoil).

**Fig. 4:**
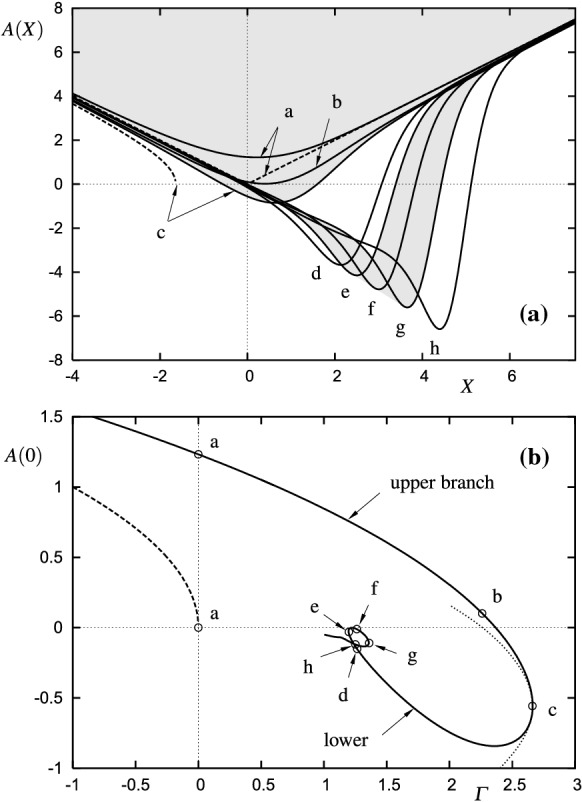
**a** Steady wall shear distributions according to (3) for unforced flow $h=v_\mathrm{w}=0$. Dashed lines: local solutions of classical boundary layer theory $A=\sqrt{X^2-\varGamma }$, cf. Fig. 3a. **b** Fundamental curve of marginal separation; dashed line: local solution of classical boundary layer theory (asymptote for $\varGamma \rightarrow -\infty$), dotted line: parabola approximation in the vicinity of the bifurcation point $\varGamma =\varGamma _\mathrm{c}$

The distinct value of the control parameter $\alpha =\alpha _\mathrm{c}$ is characterized by vanishing wall shear stress $\tau _\mathrm{w}=0$ at a single point $x=x_0$, referred to as the limiting case of marginal separation (indicated in red). The corresponding kink in the displacement thickness distribution, Fig. 3b, already indicates a *local* breakdown of Prandtl’s hierarchical procedure: in this limit, the pressure correction $\propto {\text{ Re }}^{-1/2}\ln |x-x_0|$ due to the displacement effect results in the asymptotic expansion for the flow ceasing to be uniformly valid. For values of $\alpha >\alpha _\mathrm{c}$, the numerical computations lead to a severe breakdown at $x_{b}<x_0$ in the form of the Goldstein square-root singularity (magenta curves), where the wall-normal velocity component locally behaves like $\propto {\text{ Re }}^{-1/2}(x_b-x)^{-1/2}$ [37, 38]. As shown by Stewartson [39], viscous–inviscid interaction theory typically is *not* capable of removing (strong) Goldstein singularities (for a counterexample see e.g. [40]) and later Sychev [41] concluded that a proper flow description must *avoid* the appearance of these strong singularities in general. For instance, free streamline (Helmholtz–Kirchhoff) theory and the concept of self-induced separation is one possible way out to describe massive flow separation from smooth surfaces in the limit as ${\text{ Re }}\rightarrow \infty$, see e.g. [42].

### Marginal separation *(stage II)*

Here, however, we are not concerned with the latter phenomenon, but rather focus on the weaker singularity in the limit $\alpha \rightarrow \alpha _\mathrm{c}$, $x\rightarrow x_0$ of incipient separation: if the local flow description enables the displacement and the thereby induced pressure to strongly interact on the same level of approximation, a self-consistent asymptotic triple-deck framework, commonly referred to as the theory of marginal separation, can be set up. It was originally introduced independently by Ruban [17, 18] and Stewartson, Smith and Kaups [19] and later generalized to include temporal dependence [20, 21], and also flow control devices. The pivotal outcome of this research is the fundamental equation

3 $$\begin{aligned} A^2-X^2+{\varGamma } = \displaystyle \lambda \int \limits _X^\infty \frac{\partial ^2(A-h)/\partial \xi ^2}{(\xi -X)^{1/2}}\,\mathrm{d}\xi - \gamma \int \limits _{-\infty }^X\frac{\partial A/\partial T+v_\mathrm{w}}{(X-\xi )^{1/4}}\,\mathrm{d}\xi \end{aligned}$$

for the displacement function *A*(*X*, *T*), which is related to the boundary layer characteristics (2) through the local expansions

4 $$\begin{aligned} \delta ^*\sim \delta _0^*(x_0)-{\text{ Re }}^{-1/5}[c_1\,A(X,T)+c_2\,X]+\cdots ,\quad \tau _\mathrm{w}\sim {\text{ Re }}^{-1/5}c_3\,A(X,T)+\cdots . \end{aligned}$$

Here, $X\propto {\text{ Re }}^{1/5}(x-x_0)$ is the local coordinate in streamwise direction, ${\varGamma }\propto {\text{ Re }}^{2/5}(\alpha -\alpha _\mathrm{c})$ a measure for the deviation of the control parameter from its limiting value and $T\propto {\text{ Re }}^{-1/20}{\tilde{u}}_\infty {\tilde{t}}/{\tilde{L}}$ is the time. The effect of external disturbances is incorporated by the quantities *h*(*X*, *T*) and $v_\mathrm{w}(X,T)$, representing the shape of a surface mounted obstacle^1^ [44], and the velocity distribution in a suction slot [45], respectively. All quantities have been nondimensionalized and suitably scaled to represent *O*(1) quantities, see [43, 46] for details. Furthermore, $\lambda$ and $\gamma$ are positive constants. In (4), $\delta _0^*(x_0)$ represents the value of the displacement thickness according to classical boundary layer theory in the limit $\alpha =\alpha _\mathrm{c}$ at $x=x_0$. The values of the constants $c_1>0$, $c_2$, $c_3>0$ as well as all other constants of proportionality entering the scalings depend on the specific flow problem under consideration. Representative candidates of flow problems whose theoretical description also can be condensed into a similarity law of the form (3) include channel flows with a suction slot or a displacement body at the upper wall [13, 15, 47], smooth backward facing step flows [3], retarded boundary layer flows of dense gases [48, 49], sub- or supersonic flows past flared cylinders [50] and viscous wall jets that are forced to deflect [51].

Comprehensive investigations of (3) for steady as well as unsteady, forced and unforced flows led to the following important conclusions, see Fig. 4: weak Goldstein singularities, represented by ${\varGamma }>0$, are regularized by means of the interaction strategy and may result in localized reverse flow zones characterized by $A<0$, whereas for ${\varGamma }\rightarrow \infty$ no (real) solution can be obtained, reflecting Stewartson’s finding [39]. Consequently, there exists an upper bound $\varGamma _\mathrm{c}$ for admissible steady solutions: ${\varGamma }\in (-\infty ,{\varGamma }_\mathrm{c}]$. Furthermore, the (steady, unforced flow) solutions, most conveniently depicted in the ‘parameter space’ $A(X=0)$ versus $\varGamma$, Fig. 4b, bifurcate three times (at points/curves labelled c, e and g) and therefore are (multiple) nonunique in the range ${\varGamma }\in [0,{\varGamma }_\mathrm{c})$ [19, 52]. A bifurcation analysis based on the limit process as ${\varGamma }-{\varGamma }_\mathrm{c}\rightarrow 0^\pm$ leads to a reduction of (3) to a nonlinear differential equation of Fisher–KPP type whose specific characteristics indicate a significant change of the stability behaviour at the bifurcation points [32, 53, 54]: upper/lower branch solutions are stable/unstable in general and this allows for the distinction between *short* and *long* separation bubbles (grey toned areas in Fig. 4a represent the stable range), in qualitative agreement with experimental findings. Moreover, the critical value $\varGamma _\mathrm{c}$ may effectively be altered by means of suitably chosen passive flow control devices *h*(*X*) and/or $v_\mathrm{w}(X)$, [43, 53]. In case of time-harmonic forcing, the theory reveals what has been observed numerically as well as experimentally, cf. [55, 56]: the higher the forcing frequency of the control device, the higher the stabilizing effect on the separation bubble, see [57] for details.

The solution of the initial value problem for equation (3) induced by unsteady forcing $(h,v_\mathrm{w})$ and appropriate initial conditions shows the following characteristics: subcritical flows ${\varGamma }<{\varGamma }_\mathrm{c}$ may remain bounded $|A(X,T)|<\infty$ or blow up, i.e. develop singularities, within finite time (dependent on device location, forcing amplitude and frequency), whereas supercritical flows ${\varGamma }>{\varGamma }_\mathrm{c}$ invariably lead to finite-time blow-up(s) [21, 57]. These observations, which essentially rest on the nonuniqueness of the steady two-dimensional base flow and its saddle-node bifurcation at $\varGamma _\mathrm{c}$, Fig. 4b, allow the formulation of a precise *vortex shedding criterion*: subcritical flows require a certain perturbation amplitude to trigger bubble bursting, i.e. the (repeated) release of a vortex, cf. Fig. 1. In sharp contrast, supercritical conditions are always accompanied by self-sustained vortex shedding, even in the absence of any perturbations.

As pointed out by Ryzhov and Smith [27], the Cauchy problem associated with (3) is *ill-posed*, i.e. the solution (scheme) is prone to short-scale instabilities. However, the study by Braun and Scheichl [28] shows that the application of a *composite* asymptotic model which includes higher order effects (such as e.g. the streamline curvature) successfully leads to a corresponding regularization.

A distinct feature of bubble bursting and likewise an important ingredient of the laminar–turbulent transition process is the tendency of the flow to develop progressively shorter scales in time and space. In the present context we consequently focus on solutions which lead to finite-time singularities since it is solely these which enable the scale transformations necessary for an asymptotic description. Interestingly, any of the above-mentioned blow-up solutions ultimately approaches a unique, self-similar structure, entirely independently of the choice of initial data, below or above critical flow conditions (${\varGamma }\lessgtr {\varGamma }_\mathrm{c}$), and, if present at all, the type of forcing [58]. The finite-difference scheme used to compute the spatio-temporal evolution, described in [58], turned out to suppress short-scale instabilities without any specific regularization measures. In the final phase of the calculation, the solution develops large amplitudes, which become more and more pronounced in a region of decreasingly spatial extent. Eventually, the numerical results suggest the occurrence of a singularity at a single point $X=X_\mathrm{s}$, at the blow-up time $T=T_\mathrm{s}$ in accordance with the predicted scalings of the terminal structure

5 $$\begin{aligned} A(X,T)\sim (T_\mathrm{s}-T)^{-2/3}{{\hat{A}}}_1({{\hat{X}}})+\cdots ,\quad X-X_\mathrm{s}=(T_\mathrm{s}-T)^{4/9}{{\hat{X}}}, \end{aligned}$$

as $X-X_\mathrm{s}\rightarrow 0$, $T_\mathrm{s}-T\rightarrow 0^+$ [21]. Here the blow-up profile ${{\hat{A}}}_1({{\hat{X}}})$ is governed by the homogeneous ordinary nonlinear integro-differential equation

6 $$\begin{aligned} {{\hat{A}}}_1^2=\lambda \int \limits _{{{\hat{X}}}}^\infty \frac{{{\hat{A}}}_1^{\prime \prime }}{(\xi -{{\hat{X}}})^{1/2}}\,\mathrm{d}\xi -\frac{2\gamma }{3}\int \limits _{-\infty }^{{{\hat{X}}}}\left( {{\hat{A}}}_1+\frac{2}{3}\xi {{\hat{A}}}_1^\prime \right) \frac{\mathrm{d}\xi }{({{\hat{X}}}-\xi )^{1/4}}. \end{aligned}$$

**Fig. 5:**
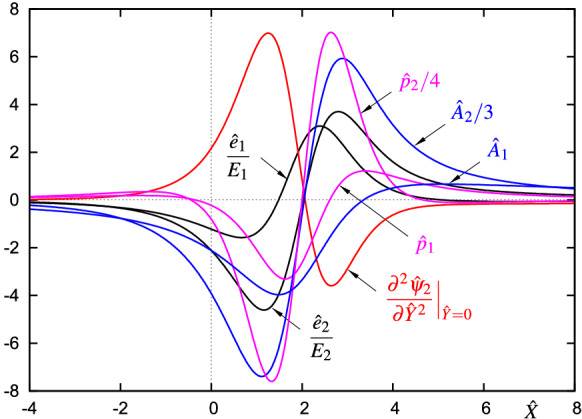
Various quantities determining the initial condition (11) of the triple-deck stage and the asymptotic behaviour of *A* and $\tau _\mathrm{w}^*$, (34) in the limit as $t\rightarrow -\infty$

Its unique nontrivial solution is depicted in Fig. 5, see also [58].

## Spike formation (*triple-deck stage III*)

As shown by Smith [21], and Elliott and Smith [29], the subsequent stage following the finite-time breakdown of marginal separation characterized by (5) and (6) is a fully nonlinear, unsteady triple-deck interaction. Its essential equation, formulated in terms of the stream function $\psi (x,y,t)$, reads

7 $$\begin{aligned} \frac{\partial ^2\psi }{\partial y\partial t}+\frac{\partial \psi }{\partial y}\frac{\partial ^2\psi }{\partial y\partial x}-\frac{\partial \psi }{\partial x}\frac{\partial ^2\psi }{\partial y^2}=-\left( 1+\frac{\partial {{\mathscr {P}}}}{\partial x}\right) +\frac{\partial ^3\psi }{\partial y^3}, \end{aligned}$$

see Appendix A for details. The induced pressure ${{\mathscr {P}}}(x,t)\sim {\text{ Re }}^{2/7}({\tilde{p}}-{\tilde{p}}_\infty )/({\tilde{\rho }}{\tilde{u}}_\infty ^2)$ and the displacement function ${{\mathscr {A}}}(x,t)$ are related to each other by the Hilbert transform, i.e. the familiar interaction law for incompressible flows, according to

8 $$\begin{aligned} {{\mathscr {P}}}=\mathscr {H}\left( \frac{\partial {{\mathscr {A}}}}{\partial x}\right) :=\frac{1}{\pi }\int \limits _{-\infty }^\infty \frac{\partial {{\mathscr {A}}}/\partial \xi }{x-\xi }\,\mathrm{d}\xi . \end{aligned}$$

Moreover, the problem is subject to the no-slip boundary conditions $\psi =\partial \psi /\partial y=0$ at the solid wall $y=0$, the matching conditions with the main part of the boundary layer

9 $$\begin{aligned} \psi \sim \frac{(y+{{\mathscr {A}}})^3}{6}+\int \limits _{-\infty }^x\frac{\partial {{\mathscr {A}}}}{\partial t}\,\mathrm{d}\xi +\frac{2}{3}\frac{{\mathscr {P}}}{y}+O(y^{-2}) \end{aligned}$$

as $y\rightarrow \infty$ and $\psi \rightarrow y^3/6$, ${{\mathscr {A}},P}\rightarrow 0$ as $|x|\rightarrow \infty$. Here $x\sim {\text{ Re }}^{2/7}{\tilde{x}}/{\tilde{L}}$, $y\sim {\text{ Re }}^{4/7}{\tilde{y}}/{\tilde{L}}$ and $t\sim {\text{ Re }}^{1/7}{\tilde{t}}{\tilde{u}}_\infty /{\tilde{L}}$ are the local coordinates in streamwise direction, wall-normal direction and the time. Again, all quantities have been suitably scaled to be of *O*(1), see Appendix A. In the triple-deck stage of the bursting event, the length and time scales are already rather short, and—in contrast to the conditions before—the induced pressure gradient $\partial {{\mathscr {P}}}/\partial x$ reaches the same order of magnitude as the imposed (adverse) pressure gradient. Moreover, the local displacement effect and the action of the wall shear stress (2) become intensified,

10 $$\begin{aligned} \delta ^*\sim \delta _0^*(x_0)-{\text{ Re }}^{-1/14}c_4\,{{\mathscr {A}}}(x,t)+\cdots ,\quad \tau _\mathrm{w}\sim {\text{ Re }}^{-1/14}c_5\,\frac{\partial ^2\psi }{\partial y^2}\Big |_{y=0}+\cdots , \end{aligned}$$

cf. (4). Here, $c_4>0$ and $c_5>0$ again denote problem specific constants.

**Fig. 6:**
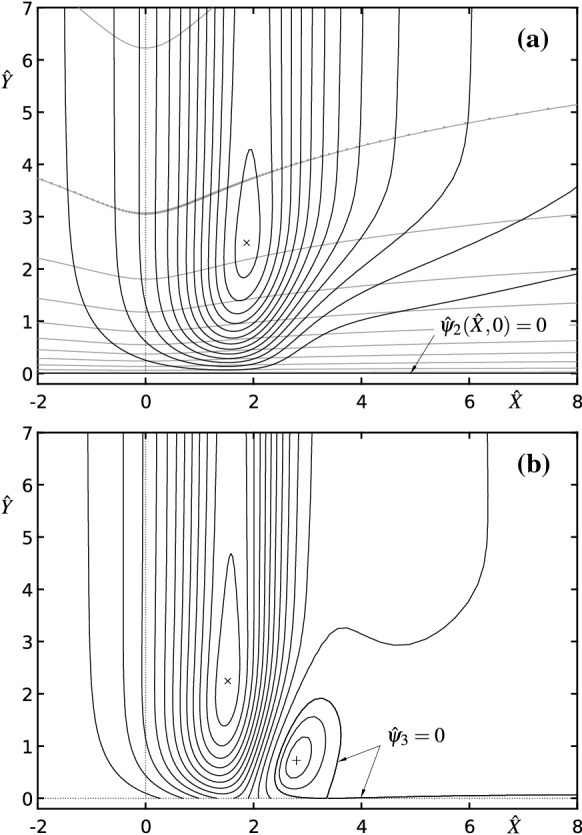
Contour plots of the perturbation stream functions (streamlines) according to (12): **a** ${\hat{\psi }}_2({\hat{X}},{\hat{Y}})$, in increments of $-0.5$ (black lines); $\times$—minimum: ${\hat{\psi }}_2(1.872,2.500)\approx -6.702$. Grey lines and full circles indicate the computational grid with $m\times n=185\times 93$ resolution and ${\hat{\eta }}={\hat{Y}}/d({\hat{X}})$ according to (16), (22); only every seventh grid line is plotted in wall-normal direction. **b** ${\hat{\psi }}_3({\hat{X}},{\hat{Y}})$, in increments of $-5$ and $+2$ (black lines); $\times$—minimum: ${\hat{\psi }}_3(1.522,2.244)\approx -62.97$, $+$—maximum: ${\hat{\psi }}_3(2.796,0.7227)\approx 5.161$

Most important, the connection of (7) to the terminal structure of (3) is ensured by specification of the initial (or equivalently matching) condition

11a $$\begin{aligned} \psi \sim \displaystyle |t|^{1/3}\left( \frac{{\hat{Y}}^3}{6}+|t|^{-7/9}{\hat{A}}\,\frac{{\hat{Y}}^2}{2}+|t|^{-14/9}{\hat{\psi }}\right) \end{aligned}$$

with the Hilbert pairs

11b $$\begin{aligned}{}[{\hat{A}},{\hat{P}}]\sim \displaystyle [{{\hat{A}}}_1,{\hat{p}}_1]({\hat{X}})+|t|^{-4/9}[{\hat{e}}_1,{\hat{p}}_{e_1}]({\hat{X}})+|t|^{-7/9}[{\hat{A}}_2,{\hat{p}}_2]({\hat{X}})+|t|^{-1}[{\hat{e}}_2,{\hat{p}}_{e_2}]({\hat{X}})+\cdots \end{aligned}$$

and

11c $$\begin{aligned} {\hat{\psi }}\sim & {} \displaystyle \left( {\hat{\psi }}_2({\hat{X}},{\hat{Y}})+{{\hat{A}}}_1^2\frac{{\hat{Y}}}{2}\right) +|t|^{-4/9}\left( {\hat{\psi }}_{e1}({\hat{X}},{\hat{Y}})+{\hat{e}}_1{\hat{A}}_1{\hat{Y}}\right) \nonumber \\&\quad \displaystyle +|t|^{-7/9}\left( {\hat{\psi }}_3({\hat{X}},{\hat{Y}})+{{\hat{A}}}_1{\hat{A}}_2{\hat{Y}}+{{\hat{A}}}_1\frac{\partial {\hat{\psi }}_2}{\partial {\hat{Y}}}+\frac{{{\hat{A}}}_1^3}{6}\right) +|t|^{-1}\left( {\hat{\psi }}_{e2}({\hat{X}},{\hat{Y}})+{\hat{e}}_2{\hat{A}}_1{\hat{Y}}\right) +\cdots \end{aligned}$$

as $t\rightarrow -\infty$. Here we have used the scalings $x=|t|^{4/9}{\hat{X}}$, $y=|t|^{1/9}{\hat{Y}}$, ${{\mathscr {A}}}=|t|^{-6/9}{\hat{A}}$ and ${{\mathscr {P}}}=|t|^{-10/9}{\hat{P}}$. For details, consult the investigation of Braun and Scheichl [28], but please note that in (11) a slightly different formulation of the stream function is used for reasons of the numerical implementation. Substitution of (11) into (7) recovers the blow-up structure of the preceding stage in form of the staggered set of boundary layer equations linearized about the sublayer separation profile ${\hat{Y}}^3/6$,

12 $$\begin{aligned} {{\mathscr {L}}}{\hat{\psi }}_i:=\frac{\partial ^3{\hat{\psi }}_i}{\partial {\hat{Y}}^3}-\frac{{\hat{Y}}^2}{2}\frac{\partial ^2{\hat{\psi }}_i}{\partial {\hat{X}}\partial {\hat{Y}}}+{\hat{Y}}\frac{\partial {\hat{\psi }}_i}{\partial {\hat{X}}}=b_i,\quad i=1,2,\ldots , \end{aligned}$$

subject to the adapted no-slip boundary conditions and the matching condition determinable from (9). Here the leading order term ${\hat{\psi }}_1={{\hat{A}}}_1{\hat{Y}}^2/2$ represents the nontrivial eigensolution of the singular operator equation ${{\mathscr {L}}}{\hat{\psi }}_1=b_1=0$ which meets all the boundary conditions. Application of the adjoint operator approach and Fredholm’s alternative as described in [28] to the second-order problem ${{\mathscr {L}}}{\hat{\psi }}_2=\frac{2}{3}{\hat{Y}}{\hat{A}}_1+\frac{4}{9}{\hat{X}}{\hat{Y}}{\hat{A}}_1^\prime +{\hat{p}}_1^\prime$ subject to the no-slip conditions ${\hat{\psi }}_2=0$, ${\hat{\psi }}_{2{\hat{Y}}}=-{\hat{A}}_1^2/2$ at ${\hat{Y}}=0$ and the interaction law ${\hat{p}}_1={{\mathscr {H}}}({\hat{A}}_1^\prime )$ eventually leads to the solvability condition (6) which determines the unique blow-up profile ${\hat{A}}_1({\hat{X}})$, Figs. 5 and 8a. The presence of the eigenfunctions ${\hat{e}}_1=E_1{{\hat{A}}}_1^\prime$, ${\hat{e}}_2=E_2({\hat{A}}_1+\frac{2}{3}{\hat{X}}{\hat{A}}_1^\prime )$ with the arbitrarily choosable amplitudes $E_1,E_2$ (reflecting the ‘history’ of the flow, or, in other words, which allows the embedding of the local flow description into a global one) shows that the spatio-temporal evolution of $\psi$ is *not* uniquely determined by the local analysis of the flow but, nevertheless, predictable. A proper start of the triple-deck computations requires the setup of an accurate numerical approximation of the time derivative term in (7) in the limit as $t\rightarrow -\infty$. To this end, the particular solution ${\hat{\psi }}_2$ to (12), chosen such that it remains bounded as ${\hat{Y}}\rightarrow \infty$, and the associated homogeneous part ${\hat{A}}_2{\hat{Y}}^2/2$ is also determined. The latter of course results from the solvability condition for the third-order problem ${{\mathscr {L}}}{\hat{\psi }}_3=\frac{13}{9}{\hat{Y}}{\hat{A}}_2+\frac{4}{9}{\hat{X}}{\hat{Y}}{\hat{A}}_2^\prime +{\hat{p}}_2^\prime +\frac{4}{3}{\hat{\psi }}_{2{\hat{Y}}}+\frac{1}{9}{\hat{Y}}{\hat{\psi }}_{2{\hat{Y}}{\hat{Y}}}+\frac{4}{9}{\hat{X}}{\hat{\psi }}_{2{\hat{X}}{\hat{Y}}}$ with ${\hat{\psi }}_3={\hat{A}}_1^3/3$, ${\hat{\psi }}_{3{\hat{Y}}}=-{\hat{A}}_1({\hat{A}}_2+{\hat{\psi }}_{2{\hat{Y}}{\hat{Y}}})$ at ${\hat{Y}}=0$. Within the scope of the present study, also the higher order terms ${\hat{\psi }}_{e1}$ and ${\hat{\psi }}_3$ were determined (similarly to ${\hat{\psi }}_2$), see Figs. 5 and 6a, b.

With these prerequisites at hand we turn to the main objective of the present work, namely the presentation of our numerical approach for solving the initial value problem (7)–(12).

### Numerical treatment of the triple-deck stage

The numerical method for solving the problem (7)–(12) comprises several components and is structured as follows.

*Spatial discretization and differentiation.* Initial attempts to approximate the spatial derivatives with a finite-difference scheme proved to be totally unreliable in terms of accuracy, especially regarding the derivatives of second and third order in the wall-normal direction. A significant increase in accuracy was achieved by the application of a spectral collocation method based on Chebyshev polynomials both in wall-normal and streamwise direction. The use of (pseudo-)spectral methods for the solution of triple-deck problems, especially in the (nonlinear) flow separation regime, originally goes back to the work of Burggraf and Duck [59], see also [33, 60]. We essentially follow the comfortable differentiation matrices approach introduced by Gajjar [61] for the treatment of viscous–inviscid interaction problems. To this end, the (semi-)infinite physical domains are mapped to the Chebyshev domain $s\in [-1,1]$, see [62, 63]. Specifically, any (unknown) field variable *f* is interpolated by a polynomial *p*(*s*) in Lagrange form, where the basis polynomials $\ell _j(s)$ are represented by the barycentric formula, see the very instructive article by Berrut and Trefethen [64] for details:

13 $$\begin{aligned} p(s)=\sum _{j=0}^n\ell _j(s) f_j,\quad \ell _j(s)=\frac{w_j}{s-s_j}\bigg [\sum _{k=0}^n\frac{w_k}{s-s_k}\bigg ]^{-1}. \end{aligned}$$

Here $f_j=p(s_j)$, $s_j=-\cos (j\pi /n)$ and $w_j=(-1)^j\,[1-(\delta _{j0}+\delta _{jn})/2]$ denote the intrinsic unknowns of the problem, the $n+1$ unequally spaced Gauss–Lobatto grid points (nodes) for the interpolation process to be well conditioned and the weights with indices $j=(0,...,n)$, respectively. Furthermore, we make use of the usual Kronecker delta $\delta _{ij}=1$ when $i=j$ and 0 otherwise.

Spatial differentiation of order *m* then is easily performed via differentiation matrices whose entries $D_{ij}^{(m)}$ can be computed analytically [64], e.g. for the differentiation order $m=1$

14 $$\begin{aligned} \begin{array}{c} f_i^\prime =p^\prime (s_i)=\displaystyle \sum _{j=0}^nD_{ij}^{(1)}f_j,\quad D_{ij}^{(1)}:=\ell _j^\prime (s_i),\\ D_{ij}^{(1)}=\displaystyle \frac{w_j/w_i}{s_i-s_j}\;\;\; \text{ for }\;\;i\not =j,\quad D_{ii}^{(1)}=-\sum _{j\not =i}D_{ij}^{(1)}. \end{array} \end{aligned}$$

To minimize the effects of roundoff errors, we follow [65] and compute the higher order differentiation matrix entries iteratively via

15 $$\begin{aligned} D_{ij}^{(m)}=\frac{m}{s_i-s_j}\left( \frac{w_j}{w_i}D_{ii}^{(m-1)}-D_{ij}^{(m-1)}\right) \;\; \text{ for }\;\;i\not =j,\quad D_{ii}^{(m)}=-\sum _{j\not =i}D_{ij}^{(m)}. \end{aligned}$$

In the context of numerical schemes involving Chebyshev polynomials the interested reader is referred to a variety of useful references in the open-source software project Chebfun^2^ [66–68].

**Fig. 7:**
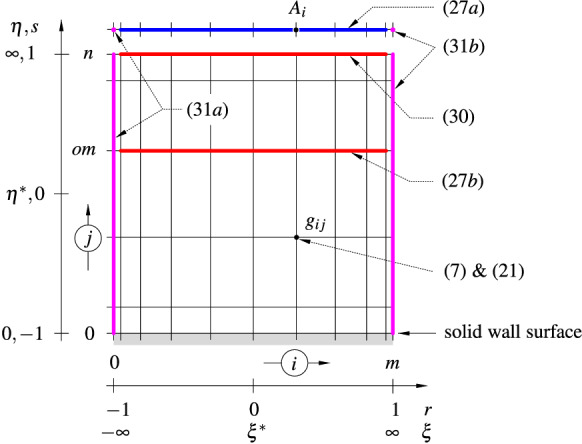
Computational domain, Gauss–Lobatto grid and allocation of the equations of the triple-deck stage

The domain mappings $\{r,s\in [-1,1]\}\rightarrow \{x\in (-\infty ,\infty ),\,y\in [0,\infty )\}$ are accomplished by means of the nonlinear transformations

16 $$\begin{aligned} x(r)=x^*+B\tan \left( \frac{\pi r}{2}\right) ,\quad y(s)=y^*+C\tan \left[ \frac{\pi (s+1)}{4}+\frac{(s-1)}{2}\arctan \left( \frac{y^*}{C}\right) \right] , \end{aligned}$$

where $x^*,y^*$ and *B*, *C* denote appropriate origin shifts and positive stretching parameters, respectively; see Fig. 7 and Table 1 for the typical numerical parameter settings. As a consequence, the elements of the differentiation matrices ${{\mathscr {D}}}_{ij}^{(m)}$ in the physical space, e.g. for the wall-normal direction, are then given by application of the chain rule (Faà di Bruno’s formula)

17 $$\begin{aligned} {{\mathscr {D}}}_{ij}^{(1)}=s_y D_{ij}^{(1)},\quad {{\mathscr {D}}}_{ij}^{(2)}=s_{yy} D_{ij}^{(1)} + s_y^2 D_{ij}^{(2)}, {{\mathscr {D}}}_{ij}^{(3)}=s_{yyy} D_{ij}^{(1)} + 3s_ys_{yy}\,\, D_{ij}^{(2)} + s_y^3 D_{ij}^{(3)}. \end{aligned}$$

Here the subscripts on *s* denote derivatives with respect to *y*, e.g.

$$\begin{aligned} s_y=\frac{\mathrm{d}s}{\mathrm{d}y}\,(s_i)=4C/\left[ \pi +2\arctan (y^*/C)\right] /\left[ C^2+(y(s_i)-y^*)^2\right] ,\quad \text{ etc }. \end{aligned}$$

*Spatial integration.* In a similar fashion, a matrix–vector notation is also used for integration, albeit the determination of the integration weights (matrix entries) requires numerical evaluation in general. Here, the aim is to apply the above introduced polynomial representation (13) of a field quantity to the Weyl fractional integral operators appearing e.g. in the equation of the blow-up profile (6). To this end, integration by parts is performed in order to ensure integrability, i.e.

$$\begin{aligned} J(x_i):=\int \limits _{-\infty }^{x_i}\!\frac{{\hat{p}}(\xi )\,\mathrm{d}\xi }{(x_i-\xi )^\sigma }=\frac{1}{1-\sigma }\!\int \limits _{-\infty }^{x_i}\!(x_i-\xi )^{1-\sigma }{\hat{p}}^\prime (\xi )\,\mathrm{d}\xi =\frac{1}{1-\sigma }\int \limits _{-1}^{r_i}[x_i-\xi (r)]^{1-\sigma }p^\prime (r)\,\mathrm{d}r. \end{aligned}$$

Here $0<\sigma <1$ and sufficient decay ${\hat{p}}(x)\sim o(|x|^{\sigma -1})$ as $x\rightarrow \pm \infty$ is assumed. Using (13) and the convention of vanishing values of *f* at the boundaries, $f_0=f_n=0$, we finally obtain

18 $$\begin{aligned} J(x_i)=\sum _{j=1}^{n-1}{{\mathscr {J}}}_{ij}f_j,\quad {{\mathscr {J}}}_{ij}:=\frac{1}{1-\sigma }\int \limits _{-1}^{r_i}[x_i-\xi (r)]^{1-\sigma }\ell _j^\prime (r)\,\mathrm{d}r \end{aligned}$$

with $\xi (r)=x(r)$ according to (16). By analogy,

19 $$\begin{aligned} I(x_i):=\int \limits _{x_i}^\infty \frac{{\hat{p}}(\xi )\,\mathrm{d}\xi }{(\xi -x_i)^\sigma }=\sum _{j=1}^{n-1}{{\mathscr {I}}}_{ij}f_j,\quad {{\mathscr {I}}}_{ij}:=\frac{1}{\sigma -1}\int \limits _{r_i}^{1}[\xi (r)-x_i]^{1-\sigma }\ell _j^\prime (r)\,\mathrm{d}r. \end{aligned}$$

For the Hilbert transform one deduces

20 $$\begin{aligned} H(x_i):=\frac{1}{\pi }\int \limits _{-\infty }^\infty \frac{{\hat{p}}(\xi )\,\mathrm{d}\xi }{x_i-\xi }=\sum _{j=1}^{n-1}{{\mathscr {H}}}_{ij} f_j,\quad {{\mathscr {H}}}_{ij}:=\frac{1}{\pi }\int \limits _{-1}^1\ln |x_i-\xi (r)|\,\ell _j^\prime (r)\,\mathrm{d}r, \end{aligned}$$

with the symmetry property ${{\mathscr {H}}}_{n-i,n-j}=-{{\mathscr {H}}}_{ij}$. The determination of the matrix entries ${{\mathscr {J}}}_{ij}, {{\mathscr {I}}}_{ij}$ and ${{\mathscr {H}}}_{ij}$ is performed in a preprocessing step by means of numerical integration (quadrature) routines of the NAG C Library (Mark 26) suitable for highly oscillatory integrands (d01skc) and which allow for (integrable) singularities at the user-specified break-points $r_i$ (d01slc).

*Singular parts, similarity properties and temporal discretization.* In order to capture the singular behaviour of $\psi$ as $y\rightarrow \infty$, (9), and its similarity properties as $x\rightarrow \pm \infty$ and $t\rightarrow -\infty$, we write

21 $$\begin{aligned} \psi (x,y,t)=(bd)^3\left[ \frac{1}{6}\left( \eta +\frac{A}{b^7d}\right) ^3+g\right] ,\quad {{\mathscr {A}}}=b^{-6}A,\quad {{\mathscr {P}}}=b^{-10}P, \end{aligned}$$

where $x=b^4\xi$, $y=bd\,\eta$, $t=\tau$ and solve for the (bounded) intrinsic unknowns $A(\xi ,\tau )$, $P(\xi ,\tau )$ and $g(\xi ,\eta ,\tau )$ in a fully implicit manner. The thereby used scaling functions

22 $$\begin{aligned} b(\tau )=\left( 1-\tau /2+\sqrt{1+\tau ^2/4}\right) ^{1/9},\quad d(\xi )=\left( 1+\xi ^2\right) ^{1/8} \end{aligned}$$

have the properties $b\sim |\tau |^{1/9}$ as $\tau \rightarrow -\infty$, $b\sim O(1)$ when $\tau \sim O(1)$ and $d\sim |\xi |^{1/4}$ as $|\xi |\rightarrow \infty$, $d\sim O(1)$ when $\xi =O(1)$. Moreover, the time derivative is replaced by a backward finite-difference approximation of second- or third-order accuracy such that adaptive time stepping on a mapped domain $\tau \in (-\infty ,\infty )\rightarrow {\bar{\tau }}\in [-1,1]$ is possible [58],

23 $$\begin{aligned} \tau ({\bar{\tau }})=D\tan \left( \frac{\pi {\bar{\tau }}}{2}\right) . \end{aligned}$$

Again, here $D>0$ denotes a stretching parameter, typically of *O*(1), table 1. However, for the asymptotic regime of large negative times $-\tau \gg 1$ finite differencing, since derived from truncated Taylor series expansions, is inappropriate and hence the time derivative for the first couple of time steps is computed analytically based on the ‘matching-in-time’ condition (11). Using the relations $\xi =|\tau |^{4/9}b^{-4}{\hat{X}}$, $A=|\tau |^{-6/9}b^6{\hat{A}}$, etc., cf. (11), one obtains e.g. for $A(\xi ,\tau )$

24 $$\begin{aligned} \frac{\partial A}{\partial \tau }\sim \frac{b^6}{|\tau |^{6/9}}\left[ 6\left( \frac{1}{b}\frac{\mathrm{d}b}{\mathrm{d}\tau }-\frac{1}{9\tau }\right) \left( {\hat{A}}+\frac{2}{3}{\hat{X}}{\hat{A}}^\prime \right) +\dot{{\hat{A}}}\right] \end{aligned}$$

as $\tau \rightarrow -\infty$. Here ${\hat{A}}^\prime \sim {\hat{A}}_1^\prime +|\tau |^{-4/9}{\hat{e}}_1^\prime +\cdots$, $\dot{{\hat{A}}}\sim -\tau ^{-1}(4|\tau |^{-4/9}{\hat{e}}_1/9+\cdots )$ and the function values of ${\hat{A}}_1({\hat{X}})$, ${\hat{e}}_1({\hat{X}})$, etc. at the temporally fixed grid points $\xi _i$ (cf. Fig. 7) are determined by means of Chebyshev interpolation in each time step. A similar relation is applied for the time derivative of $g(\xi ,\eta ,\tau )$, see [69] for details.

*Algebraic decay.* The simple application of the above-introduced differentiation and integration matrices in some way obfuscates the properties of the underlying interpolation polynomials. If the Chebyshev polynomials $T_n(r)$ of the first kind and degree $n=0,1,2,\ldots$ are combined with the transformation (16) for the streamwise coordinate, then expanding for large |*x*| yields

25 $$\begin{aligned} T_n(r(x))\sim (\pm 1)^n\left[ 1\mp \frac{2n^2B}{\pi x}+\frac{2n^2B[(n^2-1)B\mp 3\pi x^*]}{3\pi ^2x^2}+O(x^{-3})\right] ,\quad x\rightarrow \pm \infty , \end{aligned}$$

i.e. algebraic decay behaviour with negative integer powers of *x*. The unknown field quantities displacement function *A*, pressure *P* and perturbation stream function *g* indeed decay algebraically to zero (or a bounded value) in the far field, but not necessarily with a negative integer power of the respective coordinate. The quantity concerned, say *f*(*x*), therefore is written as a product [70],

26 $$\begin{aligned} f(x)=f_p(x)\,f_a(x), \end{aligned}$$

where $f_p$ is the unknown part to be interpolated and a weighting function $f_a$ to be specified such that the decay of $f_p\sim O(|x|^{-k^\pm })$ as $x\rightarrow \pm \infty$ is sufficiently strong with $k^\pm \in {\mathbb {N}}^+$. In practice, however, the decay of *f* may not be known (exactly) or may change with time. In this case, the specific far-field properties of the weighting function $f_a$ are adjusted by numerical experiments such that satisfactory results are obtained.

*Boundary conditions and implementation of equations.* After application of (21), the modified no-slip boundary conditions at the solid wall read

27a,b $$\begin{aligned}&\eta =0:\quad g=-\frac{A^3}{6d^3b^{21}},\quad \frac{\partial g}{\partial \eta }=-\frac{A^2}{2d^2b^{14}}.&\end{aligned}$$

From the far-field condition (9) it follows that $g\sim g_0(\xi ,\tau )+g_1(\xi ,\tau )/\eta +\cdots$ as $\eta \rightarrow \infty$, where

28 $$\begin{aligned} -b\,\frac{\partial A}{\partial \tau }+6\frac{\mathrm{d}b}{\mathrm{d}\tau }\left( A+\frac{2}{3}\xi \,\frac{\partial A}{\partial \xi }\right) +b^6d^2\left( 3\frac{\mathrm{d}d}{\mathrm{d}\xi } g_0+d\,\frac{\partial g_0}{\partial \xi }\right) =0, \end{aligned}$$

and

29 $$\begin{aligned} g_1=\frac{2P}{3d^4b^{14}}. \end{aligned}$$

On writing $\partial g/\partial \eta =(\partial g/\partial s)(\mathrm{d}s/\mathrm{d}\eta )$ and using the mapping (16) for the wall-normal coordinate $\eta$, one obtains the exact relation

30 $$\begin{aligned} \eta \rightarrow \infty :\quad P=-\frac{6Cd^4b^{14}}{\left[ \pi +2\arctan (\eta ^*/C)\right] }\,\frac{\partial g}{\partial s}\Big |_{s=1}={{\mathscr {H}}}\left( \frac{\partial A}{\partial \xi }\right) \end{aligned}$$

for the interaction pressure *P*. Here we want to point out that for the first derivative the differentiation matrix $D_{ij}^{(1)}$ defined by (14) on the original Chebyshev interval has to be used. For more details see Appendix B, in which a test example (Blasius boundary layer flow) serves to demonstrate the spectral method on an unbounded domain. Moreover, in (30) the interaction law (8) is used to eliminate the unknown variable *P*. Since *A* and *g* decay to zero as $x\rightarrow \pm \infty$, and to account for the elliptic character of the Hilbert transform (8), we set

31a,b $$\begin{aligned} x=-\infty :\quad g=A=0,\qquad x=\infty :\quad \frac{\partial g}{\partial r}\Big |_{r=1}=\frac{\partial A}{\partial r}\Big |_{r=1}=0. \end{aligned}$$

The assignment of the individual equations to the grid points $(\xi _i,\eta _j)$, $i=0,1,\ldots ,m$, $j=0,1,\ldots ,n$ of the computational domain for the determination of the $(m+1)\times (n+2)$ unknowns displacement function $A_i=A(\xi _i,\tau )$ and stream function $g_{ij}=g(\xi _i,\eta _j,\tau )$ at each time step can be seen in Fig. 7. The use of $m\approx 2n$ has proven advantageous in practice. Obviously, the equation of motion (7)—in its via (21) transformed version—cannot be evaluated at the upper edge $\eta \rightarrow \infty$, $j=n$; instead, the far-field condition (30) is prescribed there. In order to ensure the fulfilment of the boundary condition (27$b$), the implementation of (7) is omitted at a more or less arbitrarily selected vertical position $j=om$. The entire nonlinear algebraic system of equations is solved in a fully implicit manner with the NAG^3^ c05qbc routine in each time step. On the computer platform used (Intel^®^ Xeon^®^ CPU E5-2680 v3 @ $2.50\,\mathrm{GHz}$, up to 16 threads), the computing time for a single time step with a grid resolution of $m\times n=250\times 125$ is initially (i.e. for large negative times) about $180\,\mathrm{min}$, towards the end of the computation (before the suspected singularity occurs and the scheme no longer converges at all) up to about $450\,\mathrm{min}$ with a maximal memory usage of $\sim 14\,\mathrm{GB}$. The full computation (typically $\sim 40$ time steps) for the mentioned resolution takes approximately 1400 hours CPU time (effectively $\sim 125$ hours on our server).

*Reliability and consistency checks of the numerical solution.* The following quantitative criteria are used to evaluate and monitor the quality of the solutions found in each time step. An obvious test is to determine the maximum absolute values of the residuals (res) of the (transformed and) discretized versions of equation (7) omitted at the vertical position $j=om$ and of (28) at $\xi =\xi _i$, $i=1,2,\ldots ,m-1$, respectively:

32 $$\begin{aligned} R_1(\tau ):=\max \{|\mathrm{res}(7)(\xi _i,\eta _{om},\tau )|\},\quad R_2(\tau ):=\max \{|\mathrm{res}(28)(\xi _i,\tau )|\}. \end{aligned}$$

Typical temporal evolutions of $R_1$ and $R_2$ for different spatial resolutions are depicted in Figs. 10 and 16 . Sufficiently small values of $R_1$ and $R_2$, say less than $\approx 10^{-2}$, are required for an acceptable solution, but the decisive numerical solution acceptance criterion is derived from the assessment of the decay behaviour of the amplitude spectrum of relevant quantities at the high wave number regime. The amplitude spectrum $\breve{f}_k$—i.e. the magnitudes of the Chebyshev expansion coefficients—of the concerning approximated function *f* is given by

33 $$\begin{aligned} \breve{f}_k=\frac{2}{c_k n}\sum _{j=0}^n\frac{f_j}{c_j}\cos \left( \frac{j\pi k}{n}\right) , \quad k=0,1,\ldots ,n, \end{aligned}$$

where $c_i=2$ for $i=\{0,n\}$ and $c_i=1$ else, see e.g. [71]. An alternative method to efficiently compute the coefficients $\breve{f}_k$ is to use the discrete cosine transform (DCT). The selected spatial resolution of the numerical method (maximum polynomial degrees *m*, *n*) is considered satisfactory if the amplitudes of the quantities sought, e.g. $\breve{A}_k(\tau )$, $\breve{P}_k(\tau )$, etc., decrease sufficiently with increasing ‘wave numbers’ *k*; the corresponding quantity is then referred to as ‘fully resolved’. Typical amplitude spectra are displayed in Figs. 11 and 17 : a resolution that at first is sufficient to compute the spatio-temporal solution behaviour in the initial phase ultimately always proves to be insufficient when a blow-up event is emerging.

### Numerical results

**Fig. 8:**
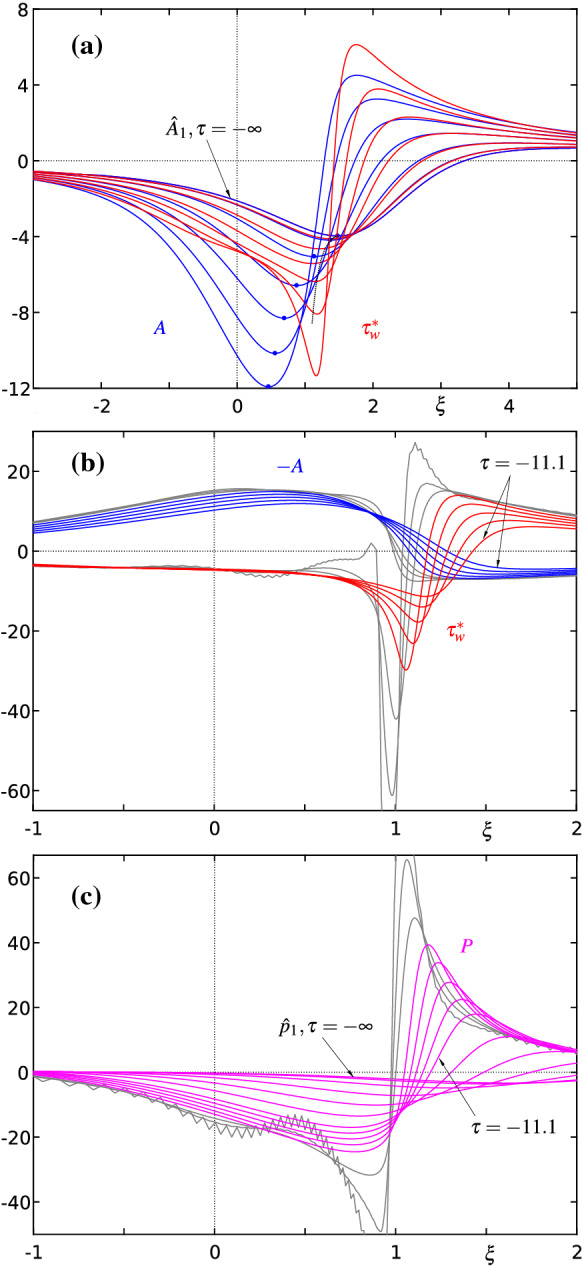
**a** Evolution of the displacement function $A(\xi ,\tau )$ (blue) and the scaled wall shear stress $\tau _\mathrm{w}^*(\xi ,\tau )$ (red) according to (7)–(31) for $\tau =-(\infty , 357, 58.2, 25.4, 17.0, 13.2, 11.1)$; each minimum location of *A* is denoted by a full circle, its asymptotic prediction (35) by a dotted line. **b** Continuation of (**a**) for $\tau =-(11.1, 10.4, 9.75, 9.26, 8.83)$. Grey lines depict the results for $\tau =-(8.50, 8.38, 8.29)$ where the used resolution $m\times n=230\times 115$ fails to meet the imposed accuracy requirements, cf. Figs. 10 and 11. **c** Corresponding interaction pressure $P(\xi ,\tau )$ (magenta) for $\tau$ of (**a**) and (**b**)

A representative numerical solution of (7)–(31) based on the proven parameter settings listed in Table 1 is depicted in Fig. 8: from (4) it is evident that the blow-up profile ${\hat{A}}_1$ is the starting form as $\tau \rightarrow -\infty$ for both the displacement function *A* and the (scaled) wall shear stress $\tau _\mathrm{w}^*$, cf. (10),

34 $$\begin{aligned} \begin{array}{l} A\sim {\hat{A}}_1+|\tau |^{-4/9}{\hat{e}}_1+|\tau |^{-7/9}{\hat{A}}_2+\cdots ,\quad g\sim \displaystyle |\tau |^{-14/9}\frac{{\hat{\psi }}_2}{d^3}+\cdots ,\\ \tau _\mathrm{w}^*:=\displaystyle b^6\frac{\partial ^2\psi }{\partial y^2}\Big |_{y=0}=A+b^7d\,\frac{\partial ^2 g}{\partial \eta ^2}\Big |_{\eta =0},\\ \tau _\mathrm{w}^*\sim \displaystyle {\hat{A}}_1+|\tau |^{-4/9}{\hat{e}}_1+|\tau |^{-7/9}\bigg ({\hat{A}}_2+\frac{\partial ^2{\hat{\psi }}_2}{\partial {\hat{Y}}^2}\Big |_{{\hat{Y}}=0}\bigg )+\cdots . \end{array} \end{aligned}$$

**Fig. 9:**
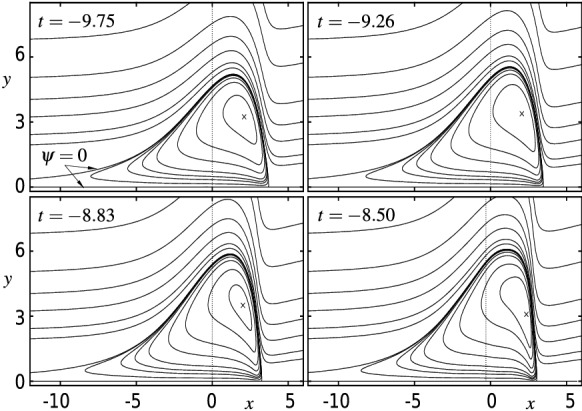
Instantaneous contour lines of the stream function $\psi (x,y,t)$ according to (7) at $t_l=-(9.75, 9.26, 8.83, 8.50)$, $l=1,2,3,4$, cf. Fig. 8b, c (see the Supplementary material for a video file). Spatial resolution $m\times n=230\times 115$; for $t_4=-8.50$, the solution no longer meets the accuracy requirements. Isolines: $\psi =(-3, -2, -1, -0.5, -0.2, -0.1, -0.05, -0.01, 0, 1, 2, 5, 10, 20, 50, 100)$. $\times$—location $(x_m,y_m)$ of minimum $\psi _m$ of $\psi$ at $t_l$:$(x_m, y_m; \psi _m)\approx \{(2.10,3.24-2.52),; (2.06,3.38;-2.94),; (2.02,3.50;-3.38), (2.36,3.08;-4.06)\}$

As time passes, these quantities deviate from each other, grow in amplitude and finally run into yet another finite-time blow-up scenario. Please notice that according to (10) the correction to the displacement thickness is proportional to $-A$. The thus changed presentation of the results in Fig. 8b renders their interpretation considerably easier. In particular, the close resemblance between the temporal development of the displacement profile with the spike formation in the separating streamline visible in the third picture from above in Fig. 1 becomes apparent. Figure 9 shows the corresponding development of instantaneous contours of the stream function for the terminal time steps.

**Table 1:** Standard specification of the numerical parameters (unless otherwise stated)

$\;m\;$	$\;n\approx m/2\;$	$\;om\;$	$\;B\;$	$\;x^*\;$	$\;C\;$	$\;y^*\;$	$\;D\;$	$\;E_1\;$	$\;E_2\;$
$100\ldots 250$	$50\ldots 125$	3*n*/4	0.65	0.9	0.7	0.	1.	1.	1.

**Fig. 10:**
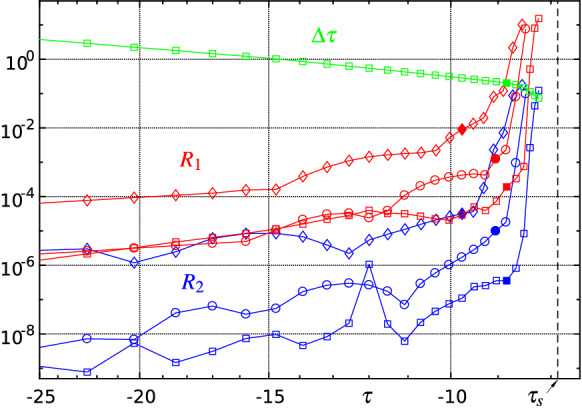
Error indicators $R_1(\tau )$ (red) and $R_2(\tau )$ (blue) according to (32) for various spatial resolutions $m\times n$: $\Diamond$—$120\times 60$, $\circ$—$180\times 90$, $\Box$—$230\times 115$. Full symbols indicate the upper limit of reliable solutions. Adaptive time step distribution $\varDelta \tau$ (green). By means of (36) estimated blow-up point: $x_\mathrm{s}\approx 0.34$, $\tau _\mathrm{s}=t_\mathrm{s}\approx -7.9$ (black dashed line)

**Fig. 11:**
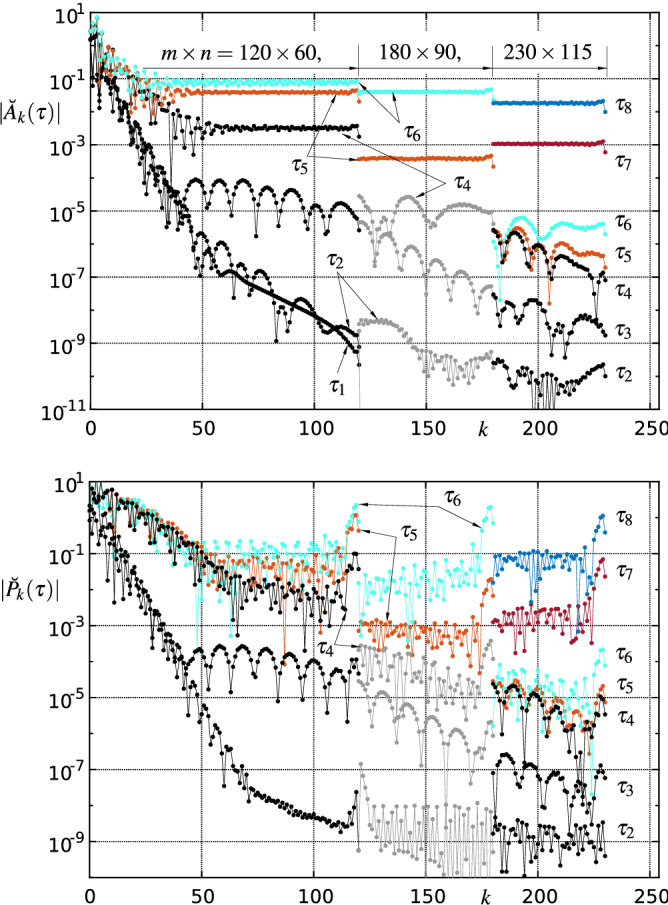
Modulus of the amplitude spectrum (33) of $A(\xi ,\tau )$ and $P(\xi ,\tau )$ for various spatial resolutions $m\times n=(120\times 60, 180\times 90, 230\times 115)$ and specific time steps $\tau _l=-(6366, 566, 17.0, 8.83, 8.65, 8.50, 8.38, 8.29)$, $l=1,\ldots ,8$. For better readability, the lower wavenumber range has been left out for the higher resolutions

It is important to ask whether every prescribed initial condition (11), which is essentially determined by specifying the amplitude values $E_1$ and $E_2$ of the eigenfunctions ${\hat{e}}_1$ and ${\hat{e}}_2$, respectively, must ultimately lead to a blow-up event. To put it the other way round: is it possible to choose $E_1$ and $E_2$ such that the evolution of $\psi$, ${{\mathscr {A}}}$ and ${{\mathscr {P}}}$ governed by (7) and (8) converges to the trivial (steady) solution $\psi =y^3/6$, ${{\mathscr {A}}}={{\mathscr {P}}}=0$ without the occurrence of any finite-time singularity? In order to gain some insight into this matter, we examine the development of the minimum of the displacement function ${\hat{A}}_\mathrm{min}$ and its location ${\hat{X}}_\mathrm{min}$ over time. We start with large negative values of time $\tau$ when (11) is valid. Setting $\partial {\hat{A}}/\partial {\hat{X}}=0$ in (11) we find

35 $$\begin{aligned} \begin{array}{l} {\hat{X}}_\mathrm{min}\sim \displaystyle {\hat{X}}_0-E_1|\tau |^{-4/9}-\frac{{\hat{A}}_2^\prime ({\hat{X}}_0)}{{\hat{A}}_1^{\prime \prime }({\hat{X}}_0)}|\tau |^{-7/9}+\frac{E_1^2{\hat{A}}_1^{\prime \prime \prime }({\hat{X}}_0)}{2{\hat{A}}_1^{\prime \prime }({\hat{X}}_0)}|\tau |^{-8/9}-\frac{2E_2{\hat{X}}_0}{3}|\tau |^{-9/9}+\cdots ,\\ {\hat{A}}_\mathrm{min}\sim \displaystyle {\hat{A}}_1({\hat{X}}_0)+{\hat{A}}_2({\hat{X}}_0)|\tau |^{-7/9}-\frac{E_1^2{\hat{A}}_1^{\prime \prime }({\hat{X}}_0)}{2}|\tau |^{-8/9}+E_2{\hat{A}}_1({\hat{X}}_0)|\tau |^{-9/9}+\cdots , \end{array} \end{aligned}$$

as $\tau \rightarrow -\infty$. Here the specific numerical values of the involved quantities are ${\hat{X}}_0\approx 1.4819$, ${\hat{A}}_1({\hat{X}}_0)\approx -3.9662$, ${\hat{A}}_1^\prime ({\hat{X}}_0)=0$, ${\hat{A}}_1^{\prime \prime }({\hat{X}}_0)\approx 4.287$, ${\hat{A}}_1^{\prime \prime \prime }({\hat{X}}_0)\approx 4.8$, ${\hat{A}}_2({\hat{X}}_0)\approx -18.660$ and ${\hat{A}}_2^\prime ({\hat{X}}_0)\approx 20.10$, cf. Figs. 5 and 8a. The inspection of (35) shows that all expansion coefficients for ${\hat{A}}_\mathrm{min}$ up to $O(|\tau |^{-8/9})$ are negative regardless of the sign of $E_1$. It, therefore, seems impossible that even large negative values of $E_2$ at $O(|\tau |^{-1})$ can change anything in the pronounced focussing tendency of the displacement function which is dominated by the leading order terms.

Eventually, one can ask whether the inclusion of properly chosen flow control measures (e.g. surface mounted obstacles of height of $O({\text{ Re }}^{-4/7})$ and/or suction slots with wall-normal velocity component of $O({\text{ Re }}^{-3/7})$) can avoid the otherwise (most likely) inevitable finite-time breakdown of the triple-deck formulation (7). This task has not yet been investigated.

### Finite-time blow-up

The currently manageable maximum resolution in space is a limiting factor. It causes the computation to terminate prior to the entry into the asymptotic blow-up regime, described below, due to the occurrence of spurious oscillations (‘wiggles’). They can definitely be ruled out as being a result of short-scale instabilities, their appearance has to be attributed to the limited spatial resolution. The finer the grid is chosen, the later the numerical solution starts to oscillate, in case of short-scale instabilities, the reverse would happen. In particular, inspection of the modulus of the amplitude spectrum $|A_k(\tau )|$, Fig. 11, shows that the individual three different spatial resolutions $m\times n=(120\times 60, 180\times 90, 230\times 115)$ yield reliable solutions up to $\tau _3$. However, up to time $\tau _4$ it is only possible to find an acceptable solution with the finest grid $m\times n=230\times 115$. To accurately predict the solution behaviour for $\tau >\tau _4$ even the finest resolution is no longer sufficient.

Nevertheless, the present results heavily underpin the similarity structure put forward in [29],

36 $$\begin{aligned}&\psi (x,y,t)\sim (t_\mathrm{s}-t)^{-3/5}{\hat{\psi }}({\hat{x}},{\hat{y}})+\cdots ,\nonumber \\&{{\mathscr {A}}}(x,t)\sim (t_\mathrm{s}-t)^{-1/5}\hat{{\mathscr {A}}}({\hat{x}})+\cdots ,\quad {{\mathscr {P}}}(x,t)\sim (t_\mathrm{s}-t)^{-4/5}\hat{{\mathscr {P}}}({\hat{x}})+\cdots \nonumber \\&\quad x-x_\mathrm{s}=(t_\mathrm{s}-t)^{3/5}{\hat{x}},\quad y=(t_\mathrm{s}-t)^{-1/5}{\hat{y}}, \end{aligned}$$

as the blow-up point $x-x_\mathrm{s}\rightarrow 0$, $t_\mathrm{s}-t\rightarrow 0^+$ is approached. The interaction relation between $\hat{{\mathscr {A}}}$ and $\hat{{\mathscr {P}}}$ reads as in (8) and the blow-up profiles ${\hat{\psi }},\hat{{\mathscr {A}}}$ and $\hat{{\mathscr {P}}}$ are governed by

37 $$\begin{aligned} \frac{2}{5}\frac{\partial {\hat{\psi }}}{\partial {\hat{y}}}+\frac{3}{5}{\hat{x}}\frac{\partial ^2{\hat{\psi }}}{\partial {\hat{y}}\partial {\hat{x}}}-\frac{1}{5}{\hat{y}}\frac{\partial ^2{\hat{\psi }}}{\partial {\hat{y}}^2}+\frac{\partial {\hat{\psi }}}{\partial {\hat{y}}}\frac{\partial ^2{\hat{\psi }}}{\partial {\hat{y}}\partial {\hat{x}}}-\frac{\partial {\hat{\psi }}}{\partial {\hat{x}}}\frac{\partial ^2{\hat{\psi }}}{\partial {\hat{y}}^2}=-\frac{\mathrm{d}\hat{{\mathscr {P}}}}{\mathrm{d}{\hat{x}}},\quad \hat{{\mathscr {P}}}={{\mathscr {H}}}\bigg (\frac{\mathrm{d}\hat{{\mathscr {A}}}}{\mathrm{d}{\hat{x}}}\bigg ) \end{aligned}$$

subject to ${\hat{\psi }}\sim \hat{{\mathscr {B}}}({\hat{x}}){\hat{y}}+\cdots$ as ${\hat{y}}\rightarrow 0$, ${\hat{\psi }}\sim ({\hat{y}}+\hat{{\mathscr {A}}})^3/6+\cdots$ as ${\hat{y}}\rightarrow \infty$. Interestingly, this terminal structure is *inviscid* in nature and therefore a (passive) viscous sublayer is required such that the no-slip condition at the solid wall can be fulfilled [29]. Introducing the sublayer scalings

38 $$\begin{aligned} \psi (x,y,t)\sim (t_\mathrm{s}-t)^{1/10}{\hat{\varPsi }}({\hat{x}},{\hat{\eta }})+\cdots ,\quad y\sim (t_\mathrm{s}-t)^{1/2}{\hat{\eta }}, \end{aligned}$$

one obtains the modified boundary layer equation

39 $$\begin{aligned} \frac{2}{5}\frac{\partial {\hat{\varPsi }}}{\partial {\hat{\eta }}}+\frac{3}{5}{\hat{x}}\frac{\partial ^2{\hat{\varPsi }}}{\partial {\hat{\eta }}\partial {\hat{x}}}+\frac{1}{2}{\hat{\eta }}\frac{\partial ^2{\hat{\varPsi }}}{\partial {\hat{\eta }}^2}+\frac{\partial {\hat{\varPsi }}}{\partial {\hat{\eta }}}\frac{\partial ^2{\hat{\varPsi }}}{\partial {\hat{\eta }}\partial {\hat{x}}}-\frac{\partial {\hat{\varPsi }}}{\partial {\hat{x}}}\frac{\partial ^2{\hat{\varPsi }}}{\partial {\hat{\eta }}^2}=-\frac{\mathrm{d}\hat{{\mathscr {P}}}}{\mathrm{d}{\hat{x}}}+\frac{\partial ^3{\hat{\varPsi }}}{\partial {\hat{\eta }}^3} \end{aligned}$$

subject to ${\hat{\varPsi }}={\hat{\varPsi }}_{{\hat{\eta }}}=0$ at ${\hat{\eta }}=0$, ${\hat{\varPsi }}\sim \hat{{\mathscr {B}}}({\hat{x}}){\hat{\eta }}+\cdots$ as ${\hat{\eta }}\rightarrow \infty$ [70]. Here the slip velocity $\hat{{\mathscr {B}}}$ and the pressure $\hat{{\mathscr {P}}}$ are imposed and related to each other by Bernoulli’s equation $-\hat{{\mathscr {P}}}^\prime =2\hat{{\mathscr {B}}}/5+3{\hat{x}}\hat{{\mathscr {B}}}^\prime /5+\hat{{\mathscr {B}}}\hat{{\mathscr {B}}}^\prime$.

**Fig. 12:**
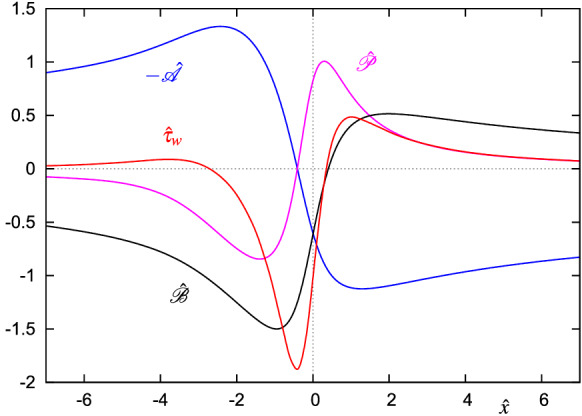
Triple-deck blow-up profiles, (37), (39): displacement function $\hat{{\mathscr {A}}}({\hat{x}})$, pressure $\hat{{\mathscr {P}}}({\hat{x}})$, slip velocity $\hat{{\mathscr {B}}}({\hat{x}})$ and wall shear stress ${\hat{\tau }}_\mathrm{w}({\hat{x}})=\partial ^2{\hat{\varPsi }}/\partial {\hat{\eta }}^2\big |_{{\hat{\eta }}=0}$. Two-parameter solution with prescribed values $\hat{{\mathscr {A}}}(0)=0.6$ and $\hat{{\mathscr {A}}}^\prime (0)=1.238$ [70]

**Fig. 13:**
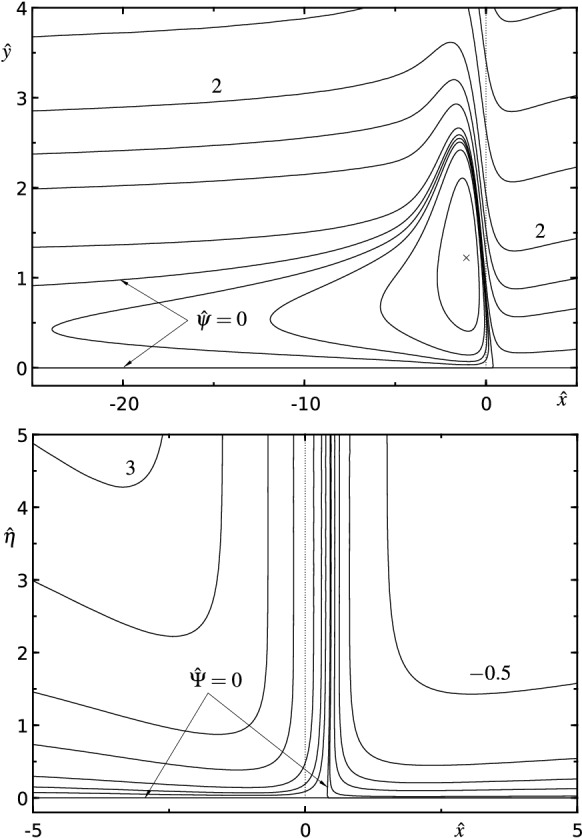
Triple-deck blow-up profiles: contour lines of **a** ${\hat{\psi }}({\hat{x}},{\hat{y}})$ according to (37); resolution $m\times n=80\times 40$, isolines ${\hat{\psi }}=(-0.5,-0.2,-0.1,0,0.1,0.5,1,2,5,10,20)$, $\times ...$ minimum $({\hat{x}}_m,{\hat{y}}_m;{\hat{\psi }}_m)\approx (-1.08,1.22;-0.873)$ and **b** ${\hat{\varPsi }}({\hat{x}},{\hat{\eta }})$ according to (39); resolution $m\times n=140\times 70$, isolines ${\hat{\varPsi }}=(-0.5,-0.2,-0.1,-0.05,-0.01,0,0.05,0.1,0.2,0.5,1,2,3)$. Two-parameter solutions with prescribed values $\hat{{\mathscr {A}}}(0)=0.6$ and $\hat{{\mathscr {A}}}^\prime (0)=1.238$ [70]

A detailed numerical analysis of (37) and (39) based on a method similar to that described above showed that the solution to (37) is not unique, but depends on two parameters, say, $\hat{{\mathscr {A}}}(0)$ and $\hat{{\mathscr {A}}}^\prime (0)$ [70]. For each set of the two parameters for which a solution to (37) could be found, the discretized version of (39) proved to be solvable as well, see Figs. 12 and 13 for a typical example. Without doubt, the solution depicted in Fig. 12 resembles features already seen in the final development of the corresponding quantities in Fig. 8b and therefore justifies the scalings (36). From a physical point of view, the terminal form (36)–(39) of the triple-deck stage indicates the emergence of a predominantly inviscid region where the crucial dynamics of the bursting process take place. Viscous effects are then confined to a narrow region adjacent to the solid wall. Nonetheless, a proper overall flow description requires a compatible viscous sublayer solution to exist.

## Treatment of the ill-posed Cauchy problem (*stage III*)

Worth mentioning is the fact that some inconsistencies associated with the proposed Cauchy problem (7), (11) were discovered in [29], and their repercussions on the solvability in general necessitate a comprehensive discussion. In that paper, the occurrence of instabilities was put in the context of an ill-posed Cauchy problem. More precisely, it was shown that the instability of the velocity field against short-scale disturbances and, entailed by that, the incorrectness of the problem are a direct consequence of the abnormal dispersion relation governing the linearized problem in the limit of very high wave numbers *k*.

To reconsider this issue, we start from the assumption that the solution to (7) near a position $x_0$ and for a short time interval $t-t_0\ll 1$ might be expanded as

40 $$\begin{aligned} \begin{array}{l} \psi = \displaystyle \psi _0(y) + \frac{\partial \psi }{\partial x}\Big |_{x_0, t_0}(x-x_0) + \frac{\partial \psi }{\partial t}\Big |_{x_0, t_0}(t-t_0) + \delta _\psi \,\psi _1(x-x_0, y,t- t_0) +\cdots ,\\ {{\mathscr {A}}} = \displaystyle {{\mathscr {A}}}_0 + \frac{\partial {{\mathscr {A}}}}{\partial x}\Big |_{x_0, t_0}(x-x_0) + \frac{\partial {{\mathscr {A}}}}{\partial t}\Big |_{x_0, t_0}(t-t_0) + \delta _{{\mathscr {A}}}\,{{\mathscr {A}}}_1 (x-x_0, t- t_0)+\cdots ,\\ {{\mathscr {P}}} = \displaystyle {{\mathscr {P}}}_0 + \frac{\partial {{\mathscr {P}}}}{\partial x}\Big |_{x_0, t_0}(x-x_0) + \frac{\partial {{\mathscr {P}}}}{\partial t}\Big |_{x_0, t_0}(t-t_0) + \delta _{{\mathscr {P}}}\,{{\mathscr {P}}}_1 (x-x_0, t- t_0)+\cdots , \end{array} \end{aligned}$$

where $\psi _0(y)$, ${{\mathscr {A}}}_0$ and ${{\mathscr {P}}}_0$ denote the concerning function values at the sampling point $(x_0,t_0)$. The additional terms $\psi _1$, ${{\mathscr {A}}}_1$ and ${{\mathscr {P}}}_1$ supplementing the Taylor series expansions of the background flow account for the emergence of possibly unstable waves with amplitudes $\delta _\psi$, $\delta _{{\mathscr {A}}}$ and $\delta _{{\mathscr {P}}}$, respectively, the sizes of which are defined below. For the description of these short-scale instabilities the distinguished limit is chosen in which a balance between pressure gradient, local time derivative and convective terms exists. Therefore the necessary scalings

41 $$\begin{aligned} t-t_0 = \alpha \, t_1,\quad x-x_0 = \alpha ^{3/5}\,x_1, \quad y=\alpha ^{-1/5} y_1,\quad \text {with}\quad \alpha \ll 1, \end{aligned}$$

can be readily obtained from (36). Here, however, the disturbances are assumed to occur prior to the blow-up structure and to be smaller in magnitude, implying the order-of-magnitude relationship

42 $$\begin{aligned} \delta _\psi =\delta _{{\mathscr {A}}}\,\alpha ^{-2/5}=\delta _{{\mathscr {P}}}\,\alpha ^{1/5}\ll \alpha ^{-3/5}. \end{aligned}$$

Recalling that $\psi _0(y)\rightarrow y^3/6$ as $y\rightarrow \infty$, one then obtains from (7) together with (40) and (41) the linear leading-order equation

43 $$\begin{aligned} \frac{\partial ^2\psi _1}{\partial y_1\partial t_1}+\frac{1}{2}y_1^2\frac{\partial ^2\psi _1}{\partial y_1\partial x_1}-y_1\frac{\partial ^2\psi _1}{\partial y_1^2}=-\frac{\partial {{{\mathscr {P}}}_1}}{\partial x_1}, \end{aligned}$$

subject to the conditions $\psi _1={{\mathscr {B}}}_1\,y_1$ as $y_1 \rightarrow 0$, $\psi _1 \rightarrow {{\mathscr {A}}}_1\,y_1^2/2$ as $y_1 \rightarrow \infty$ and ${{\mathscr {P}}}_1=\mathscr {H}({\partial {{\mathscr {A}}}_1}/{\partial x_1})$. The viscous sublayer necessary to bring the slip velocity ${{\mathscr {B}}}_1$ satisfying $\partial {{\mathscr {B}}}_1/ \partial t_1 = -\partial {{\mathscr {P}}}_1/ \partial x_1$ at rest is of thickness $O(\alpha ^{1/2})$. However, the latter problem will not be investigated in the following.

Using a Fourier approach in the sense of a stability analysis such that each quantity *f* is expressed as

44 $$\begin{aligned} f(x_1, y_1, t_1)= \text {e}^{\mathrm{i}\omega _1 t_1}\mathscr {F}^{-1}\left( \tilde{f}(k_1,y_1,\omega _1)\right) =\text {e}^{\mathrm{i}\omega _1 t_1}\frac{1}{\sqrt{2\pi }}\int _{-\infty }^\infty \tilde{f}(k_1,y_1,\omega _1)\,\text {e}^{\mathrm{i} k_1 x_1}\mathrm{d}k_1, \end{aligned}$$

where $k_1$ is real, and solving the ordinary differential equation resulting from (43) then yields

45 $$\begin{aligned} {\tilde{\psi }}_1= -\frac{\left[ z^2\,\arctan (z)+ \arctan (z) + z\right] \sqrt{2\,\omega _1\,k_1}}{2\,\omega _1}\,\tilde{{\mathscr {P}}}_1, \quad z= \frac{y_1\,k_1}{\sqrt{2\,\omega _1\,k_1}}, \end{aligned}$$

with $\sqrt{\cdot }$ understood as the principal value. In the limit as $y_1\rightarrow \infty$, ${\tilde{\psi }}_1$ behaves as

46 $$\begin{aligned} {\tilde{\psi }}_1 \rightarrow - \frac{ \pi \, y_1^2\,|k_1| \sqrt{2\,\omega _1\,k_1} }{8\, \omega _1^2}\, \tilde{{\mathscr {P}}}_1. \end{aligned}$$

Application of the constraint ${{\mathscr {P}}}_1=\mathscr {H}({\partial {{\mathscr {A}}}_1}/{\partial x_1})$, i.e. $\tilde{{\mathscr {P}}}_1= |k_1|\tilde{{\mathscr {A}}}_1$ and the far-field condition stated above gives $4\,\omega _1^2+\pi k_1^2 \sqrt{2\,\omega _1 k_1} = 0$, which, when $\omega _1$ and $k_1$ are replaced by their unscaled counterparts $\omega = \alpha ^{-1} \omega _1$ and $k=\alpha ^{-3/5}k_1$, reads

47 $$\begin{aligned} k\rightarrow \pm \infty :\quad 4\,\omega ^2+\pi \,k^2 \sqrt{2\,\omega k} \sim 0. \end{aligned}$$

This local dispersion relation for the high-wave-number limit has two complex roots, one is associated with stable and the other with unstable modes. However, as also pointed out in [29], the growth rates of the thus occurring short-scale instabilities obey $-\text{ Im }(\omega )\propto |k|^{5/3}$ and are therefore not bounded from above. Consequently, the given Cauchy problem (7), (11) turns out to be ill-posed and has to be regularized. It should be noted that the above analysis is similar to the triple-deck study of the Blasius boundary layer with respect to the so-called upper-branch stability, see [72], insofar as, to leading order, viscosity does not enter the dispersion relation. However, for the case of an attached boundary layer, the leading-order dispersion relation for high wave numbers leads to neutral modes only, whereas here the situation is obviously completely different.

### Composite asymptotic model

The essence of the above result is that higher order terms in ${\text{ Re }}^{-1}$ not included so far must play a distinctive role in the evolution of the separation process, given the bounded spectrum the unsteady Navier–Stokes equations are assumed to have, but which the leading order problem fails to deliver. However, to the authors’ knowledge, there does not exist a rigorous asymptotic theory for the regularization of otherwise ill-posed Cauchy problems. In other words, the filtering process entailed by the temporal and spatial scales the asymptotic analysis is based on may lead to the undesired result that the terms needed for regularizing the Cauchy problem at a certain level of approximation cannot be incorporated into it. They will thus enter the equations of higher order and, by that, form the higher order forcing terms. Consequently, the only way to regularize the original problem is to set up a *composite* asymptotic model, where these corrections are taken as part of the leading order problem such that they can effectively contribute to stabilizing its homogeneous solution against short-scale disturbances.

This method has been proven very successful in the past, see e.g. [73] and [74] as well as [75] and the references therein, and, in particular, for studying the unsteady marginal separation stage preceding the triple-deck stage discussed here, see [28]. As revealed in these studies, for higher order asymptotic terms to play the role of regularization terms, they must at least generate *derivatives* with respect to *t* and *x* of orders that are higher than those already present in the system. Furthermore, in [28] it was shown that the induced streamwise pressure gradient in the lower deck which replaces the original one in order to form a composite model equation has to include the effects of the streamline curvature in the main part of the boundary layer. Surprisingly, a second correction term, which is due to the unsteady nature of the main deck, was found. Whereas both appear naturally in many triple-deck problems as higher order contributions to the leading-order pressure gradient, see e.g. [72, 76, 77], only the one stemming from the streamline curvature is usually used as regularization term, [73–75, 78]. The following analysis of the triple-deck problem considered here will show that, in principle, the regularization presented in [28] can be taken over almost unchanged.

Inspection of the far-field condition of the lower-deck solution as $y\rightarrow \infty$, see (9), shows that in the main-deck region MD, where ${\bar{y}}=O(1)$, the expansion of the stream function assumes the form

48 $$\begin{aligned} \psi ({\check{x}},{\bar{y}},{\check{t}})\sim \displaystyle \varepsilon ^{7}\psi _{00}({\bar{y}}) + \varepsilon ^{8}{\bar{\psi }}_1 + \varepsilon ^{9}\,{\bar{\psi }}_2 + \varepsilon ^{10}\,{\bar{\psi }}_3 + \varepsilon ^{11}\,{\bar{\psi }}_4 +\cdots ,\quad \varepsilon :={\text{ Re }}^{-1/14}. \end{aligned}$$

Please notice that in the following, we will use the unscaled versions of the field quantities and length scales, i.e. those before the affine transformations (72) were applied. Substitution of (48) into the streamwise momentum equation yields to leading order

49 $$\begin{aligned} {{\mathscr {L}}}{\bar{\psi }}_1:=\psi _{00}'\,\frac{\partial ^2{\bar{\psi }}_1}{\partial {\check{x}}\partial {\bar{y}}}-\psi _{00}''\,\frac{\partial {\bar{\psi }}_1}{\partial {\check{x}}}= 0, \end{aligned}$$

and additionally,

50 $$\begin{aligned} \begin{array}{l} {{\mathscr {L}}}{\bar{\psi }}_2 = -\displaystyle \frac{\partial {\bar{\psi }}_1}{\partial {\bar{y}}}\frac{\partial ^2{\bar{\psi }}_1}{\partial {\check{x}}\partial {\bar{y}}}+\frac{\partial ^2{\bar{\psi }}_1}{\partial {\bar{y}}^2}\frac{\partial {\bar{\psi }}_1}{\partial {\check{x}}},\\ {{\mathscr {L}}}{\bar{\psi }}_3 = -\displaystyle \frac{\partial {\bar{\psi }}_1}{\partial {\bar{y}}}\frac{\partial ^2{\bar{\psi }}_2}{\partial {\check{x}}\partial {\bar{y}}}+\frac{\partial ^2{\bar{\psi }}_1}{\partial {\bar{y}}^2}\frac{\partial {\bar{\psi }}_2}{\partial {\check{x}}}-\displaystyle \frac{\partial {\bar{\psi }}_2}{\partial {\bar{y}}}\frac{\partial ^2{\bar{\psi }}_1}{\partial {\check{x}}\partial {\bar{y}}}+\frac{\partial ^2{\bar{\psi }}_2}{\partial {\bar{y}}^2}\frac{\partial {\bar{\psi }}_1}{\partial {\check{x}}}-\displaystyle \frac{\partial ^2{\bar{\psi }}_1}{\partial {\bar{y}}\partial {\check{t}}},\\ {{\mathscr {L}}}{\bar{\psi }}_4 = -\displaystyle \frac{\partial {\bar{\psi }}_1}{\partial {\bar{y}}}\frac{\partial ^2{\bar{\psi }}_3}{\partial {\check{x}}\partial {\bar{y}}}+\frac{\partial ^2{\bar{\psi }}_1}{\partial {\bar{y}}^2}\frac{\partial {\bar{\psi }}_3}{\partial {\check{x}}}-\displaystyle \frac{\partial {\bar{\psi }}_2}{\partial {\bar{y}}}\frac{\partial ^2{\bar{\psi }}_2}{\partial {\check{x}}\partial {\bar{y}}}+\frac{\partial ^2{\bar{\psi }}_2}{\partial {\bar{y}}^2}\frac{\partial {\bar{\psi }}_2}{\partial {\check{x}}}\\ \quad \quad \quad \quad -\displaystyle \frac{\partial {\bar{\psi }}_3}{\partial {\bar{y}}}\frac{\partial ^2{\bar{\psi }}_1}{\partial {\check{x}}\partial {\bar{y}}}+\frac{\partial ^2{\bar{\psi }}_3}{\partial {\bar{y}}^2}\frac{\partial {\bar{\psi }}_1}{\partial {\check{x}}} - p_{00} + \psi _{00}^{\prime \prime \prime }-\displaystyle \frac{\partial \bar{{\mathscr {P}}}}{\partial {\check{x}}} - \displaystyle \frac{\partial ^2{\bar{\psi }}_2}{\partial {\bar{y}}\partial {\check{t}}}. \end{array} \end{aligned}$$

The last equation in (50) is interesting insofar in that here, the induced pressure component of leading order enters. Furthermore, the pressure generated by ${\bar{\psi }}_4$ in the upper deck and acting in the lower deck will form the component of $O(\varepsilon ^{7})$ in the pressure expansion. This fact suggests a pressure variation in *transverse* direction to come into play, since the normal gradient of precisely this term of $O(\varepsilon ^{7})$ is comparable in size with the leading-order convective term in the transverse momentum equation. Consequently, the extended version of the *streamwise* pressure gradient in the main deck is given by

51 $$\begin{aligned} \frac{\partial p}{\partial {\check{x}}}\sim \varepsilon ^4\left( p_{00} + \frac{\partial \bar{{\mathscr {P}}}}{\partial {\check{x}}}\right) + \varepsilon ^5\frac{\partial \bar{{\mathscr {P}}}_2}{\partial {\check{x}}} + \varepsilon ^{6}\frac{\partial \bar{{\mathscr {P}}}_3}{\partial {\check{x}}}+\varepsilon ^{7}\frac{\partial \bar{{\mathscr {P}}}_4({\check{x}},{\bar{y}},{\check{t}})}{\partial {\check{x}}}+ \cdots , \end{aligned}$$

where all terms, except for $\bar{{\mathscr {P}}}_4({\check{x}},{\bar{y}},{\check{t}})$, are identical to the corresponding components in the lower deck. As already indicated by the above order-of-magnitude argument, the asymptotic expansion of the transverse momentum equation then leads to the relationship

52 $$\begin{aligned} \psi _{00}'\,\frac{\partial ^2{\bar{\psi }}_1}{\partial {\check{x}}^2}=\frac{\partial \bar{{\mathscr {P}}}_4}{\partial {\bar{y}}}, \end{aligned}$$

revealing the intrusion of a normal pressure gradient in the MD. As will be shown in the following, the last equation in (50) together with (52) will induce a pressure response in the lower deck that is proportional to the curvature of the streamlines, i.e. the second derivative of the displacement function $\bar{{\mathscr {A}}}$ with respect to the streamwise coordinate.

After applying the rules for matching with the lower deck and extracting the singular parts of the resulting integral, one can write the solutions to (49) and (50) as

53 $$\begin{aligned} \displaystyle \frac{\partial {\bar{\psi }}_1}{\partial {\check{x}}}= & {} \displaystyle \frac{\psi _{00}'}{p_{00}}\frac{\partial \bar{{\mathscr {A}}}}{\partial {\check{x}}},\quad \displaystyle \frac{\partial {\bar{\psi }}_2}{\partial {\check{x}}}=\displaystyle \frac{\psi _{00}''}{p_{00}^2}{\bar{{\mathscr {A}}}}\,\frac{\partial \bar{{\mathscr {A}}}}{\partial {\check{x}}},\quad \displaystyle \frac{\partial {\bar{\psi }}_3}{\partial {\check{x}}}=\displaystyle \frac{\psi _{00}'''}{2\,p_{00}^3}{\bar{{\mathscr {A}}}}^2\frac{\partial \bar{{\mathscr {A}}}}{\partial {\check{x}}}+\displaystyle \frac{1}{p_{00}}\frac{\partial \bar{{\mathscr {A}}}}{\partial {\check{t}}},\nonumber \\ \displaystyle \frac{\partial {\bar{\psi }}_4}{\partial {\check{x}}}= & {} \displaystyle \frac{\psi _{00}''''}{6\,p_{00}^4}{\bar{{\mathscr {A}}}}^3\frac{\partial \bar{{\mathscr {A}}}}{\partial {\check{x}}} +\psi _{00}'\displaystyle \left[ \int _0^{{\bar{y}}}\left( \frac{\psi _{00}'''-p_{00}}{(\psi _{00}')^2} + \frac{p_{00}}{U_{00}^2}\right) \mathrm{d}\eta -\frac{p_{00}}{U_{00}^2} {\bar{y}} \right] \nonumber \\&+\psi _{00}'\displaystyle \frac{\partial \bar{{\mathscr {P}}}}{\partial {\check{x}}}\left[ \int _0^{{\bar{y}}}\left( -\frac{1}{(\psi _{00}')^2} + \frac{4}{p_{00}^2 \eta ^4} + \frac{1}{U_{00}^2}\right) \mathrm{d}\eta + \frac{4}{3 p_{00}^2 {{\bar{y}}}^3}-\frac{{\bar{y}}}{U_{00}^2}\right] . \end{aligned}$$

Please note that for the derivation of these expressions, the expansions ${\bar{y}}\rightarrow 0:$ $\psi _{00}=p_{00} {\bar{y}}^3/6 + (2 p_{00} p_{01}/7!) {\bar{y}}^7 +\cdots$ and ${\bar{y}}\rightarrow \infty :$ $\psi _{00}=U_{00}\,{\bar{y}} + \cdots$ have been used (see e.g. [42]). In addition, the equation for the normal pressure gradient (52) results in the expression

54 $$\begin{aligned} \frac{\partial \bar{{\mathscr {P}}}_4}{\partial {\check{x}}}=\frac{\partial \check{{\mathscr {P}}}_4({\check{x}},{\check{t}})}{\partial {\check{x}}} +\frac{1}{p_{00}}\frac{\partial ^3 \bar{{\mathscr {A}}}}{\partial {\check{x}}^3}\left[ \int _0^{{\bar{y}}}\left( (\psi _{00}')^2 - {U_{00}^2}\right) \mathrm{d}\eta + U_{00}^2 {\bar{y}}\right] . \end{aligned}$$

Here, $\check{{\mathscr {P}}}_4$ denotes the induced pressure of order $O(\varepsilon ^{7})$ acting in the lower deck, which remains to be determined later.

As mentioned above, the purpose of the analysis presented here is the identification of those pressure terms that are promising candidates for regularizing the Cauchy problem stated in (7) and (11). Hence, they have to be related directly to the leading order effects represented by $\bar{{\mathscr {A}}}$. For simplicity, therefore, lower-deck displacement functions of higher order, such as $\bar{{\mathscr {A}}}_2$, $\bar{{\mathscr {A}}}_3$ and $\bar{{\mathscr {A}}}_4$, have been omitted in the main-deck solutions.

The upper deck (UD) expansions, with the appropriate coordinate normal to the wall now being ${\hat{y}}=\varepsilon ^{-3}{\bar{y}}$, are

55 $$\begin{aligned} \psi ({\check{x}},{\hat{y}},{\check{t}})\sim \varepsilon ^{4}\,U_{00}{\hat{y}} + \varepsilon ^{8}\,{\hat{\psi }}_1 + \varepsilon ^{9}\,{\hat{\psi }}_2 + \varepsilon ^{10}\,{\hat{\psi }}_3 + \varepsilon ^{11}\,{\hat{\psi }}_4+\cdots , \end{aligned}$$

and

56 $$\begin{aligned} p({\check{x}},{\hat{y}},{\check{t}})\sim (1-U_{00}^2)/2 + \varepsilon ^{4}\,(p_{00}{\check{x}} +{\hat{p}}_1) + \varepsilon ^{5}\,{\hat{p}}_2 +\varepsilon ^{6}\,{\hat{p}}_3 +\varepsilon ^{7}\,{\hat{p}}_4 + \cdots \end{aligned}$$

Here, the quantities ${\hat{p}}_j$, $j=1,2,3,4$ represent the induced pressure fields, which decay for large distances from the origin. Through substitution into the momentum equations, it is easy to verify that the terms in the stream function and the pressure expansions satisfy the linearized Euler equations

57 $$\begin{aligned} \begin{array}{l} \varDelta {\hat{p}}_1=0\quad \text {with}\quad \displaystyle \frac{\partial {\hat{p}}_1}{\partial {\hat{y}}}=\displaystyle U_{00}\frac{\partial ^2{\hat{\psi }}_1}{\partial {\check{x}}^2},\quad \varDelta {\hat{p}}_2=0\quad \text {with}\quad \displaystyle \frac{\partial {\hat{p}}_2}{\partial {\hat{y}}}=\displaystyle U_{00}\frac{\partial ^2{\hat{\psi }}_2}{\partial {\check{x}}^2},\\ \varDelta {\hat{p}}_3=0\quad \!\text {with}\!\quad \displaystyle \frac{\partial {\hat{p}}_3}{\partial {\hat{y}}}=\displaystyle \frac{\partial ^2{\hat{\psi }}_1}{\partial {\check{x}}\partial {\check{t}}}+\displaystyle U_{00}\frac{\partial ^2{\hat{\psi }}_3}{\partial {\check{x}}^2},\!\quad \varDelta {\hat{p}}_4=0\!\quad \text {with}\!\quad \displaystyle \frac{\partial {\hat{p}}_4}{\partial {\hat{y}}}=\frac{\partial ^2{\hat{\psi }}_2}{\partial {\check{x}}\partial {\check{t}}}+\displaystyle U_{00}\frac{\partial ^2{\hat{\psi }}_4}{\partial {\check{x}}^2}, \end{array} \end{aligned}$$

where $\varDelta$ is the Laplacian $\partial ^2/\partial {\check{x}}^2 + \partial ^2/\partial {\hat{y}}^2$.

In general, the solutions for the pressure fields are sought in the semi-infinite half-plane ${\hat{y}}\ge 0$ with the the relations for the wall-normal pressure gradients taken as Neumann boundary conditions at ${\hat{y}}=0$. For the calculation of the streamwise pressure gradient at ${\hat{y}}=0$, a simplified approach is possible by exploiting the fact that the derivatives $\partial {\hat{p}}_j/\partial {\hat{y}}$ and $\partial {\hat{p}}_j/\partial {\check{x}}$, $j=1,2,3,4$ satisfy the Cauchy–Riemann equations such that the complex functions $\partial {\hat{p}}_j/\partial {\hat{y}} + \mathrm{i}\,\partial {\hat{p}}_j/\partial {\check{x}}$ are analytic. As a consequence, the imaginary part of each function at ${\hat{y}}=0$ is given by the Hilbert transform (8) of the real part. Relying on the matching principles and taking into account that apart from $\bar{{\mathscr {P}}}_4$ the MD pressure terms do not depend on ${\bar{y}}$, one then obtains from (57) and (53)

58 $$\begin{aligned} \frac{\partial \bar{{\mathscr {P}}}}{\partial {\check{x}}} = \frac{U_{00}^2}{p_{00}}\,\mathscr {H}\left( \frac{\partial ^2\bar{{\mathscr {A}}}}{\partial {\check{x}}^2}\right) ,\quad \frac{\partial \bar{{\mathscr {P}}}_2}{\partial {\check{x}}}=0\quad \text {and}\quad \frac{\partial \bar{{\mathscr {P}}}_3}{\partial {\check{x}}} = 2\frac{U_{00}}{p_{00}}\,\mathscr {H}\left( \frac{\partial ^2\bar{{\mathscr {A}}}}{\partial {\check{x}}\partial {\check{t}}}\right) . \end{aligned}$$

As expected, the well-known leading order result for $\bar{{\mathscr {P}}}$ is recovered. Moreover, the last equations in (57) and (53), respectively, together with (54) lead to

59 $$\begin{aligned} \begin{array}{c} \displaystyle \frac{\partial {\hat{p}}_4}{\partial {\check{x}}}({\check{x}},{\hat{y}}=0,{\check{t}}) = -\mathscr {H}\left( \frac{\partial ^2\bar{{\mathscr {P}}}}{\partial {\check{x}}^2}\right) I_1 = \frac{\partial \check{{\mathscr {P}}}_4({\check{x}},{\check{t}})}{\partial {\check{x}}} -\frac{U_{00}^2}{p_{00}}\frac{\partial ^3\bar{{\mathscr {A}}}}{\partial {\check{x}}^3}\,I_2,\\ I_1= \displaystyle \int _0^{\infty }\left( \frac{U_{00}^2}{(\psi _{00}')^2} - \frac{4 U_{00}^2}{p_{00}^2 \eta ^4} - 1\right) \mathrm{d}\eta ,\quad I_2=\int _0^{\infty }\left( 1-\frac{(\psi _{00}')^2}{U_{00}^2}\right) \mathrm{d}\eta . \end{array} \end{aligned}$$

By invoking the relation $\mathscr {H}^2\left( f({\check{x}})\right) =-f({\check{x}})$, one can infer from (58) and (59) that the fourth component of the lower-deck pressure gradient is given by

60 $$\begin{aligned} \frac{\partial \check{{\mathscr {P}}}_4}{\partial {\check{x}}} = \frac{U_{00}^2}{p_{00}}\frac{\partial ^3\bar{{\mathscr {A}}}}{\partial {\check{x}}^3}\,(I_1 +I_2). \end{aligned}$$

Furthermore, it should be noted here that as a matter of course, the singular parts of the solutions (53) and (54) as ${\bar{y}} \rightarrow 0$ and ${\bar{y}} \rightarrow \infty$ match seamlessly with the corresponding counterparts in the lower and upper deck, respectively.

As a consequence, when the terms from (58) and (60) are combined, the affine transformations (72) are reapplied and $\varepsilon \rightarrow p_{00}^{-1/14}U_{00}^{3/14}\varepsilon$ is redefined, the induced pressure gradient replacing $\partial {{\mathscr {P}}}/\partial x$ in the lower-deck triple-deck problem (7) in order to form a *composite* model equation can be written as

61 $$\begin{aligned} \frac{\partial {{\mathscr {P}}}_\mathrm{c}}{\partial x}=\mathscr {H}\left( \frac{\partial ^2{{\mathscr {A}}}}{\partial x^2}\right) + 2\,\varepsilon ^2\,\mathscr {H}\left( \frac{\partial ^2 {{\mathscr {A}}}}{\partial x \partial t}\right) + \varepsilon ^3\,\frac{\partial ^3{{\mathscr {A}}}}{\partial x^3}\,C,\quad C=(I_1+I_2)\sqrt{\frac{p_{00}}{U_{00}}}>0. \end{aligned}$$

The constant *C* has to be calculated from the unperturbed boundary layer separation profile $\psi _{00}^\prime ({\bar{y}})=U(x_0,{\bar{y}})$ with $\psi _{00}^\prime ({\bar{y}}\rightarrow \infty )\rightarrow U_{00}$ and the prescribed pressure gradient $p_{00}>0$ at $x=x_0$. It turns out to be positive in all cases investigated, e.g. for the Falkner–Skan similarity separation profile characterized by the critical Hartree parameter $\beta \approx -0.19884$ one obtains $C\approx 0.4170$.

**Fig. 14:**
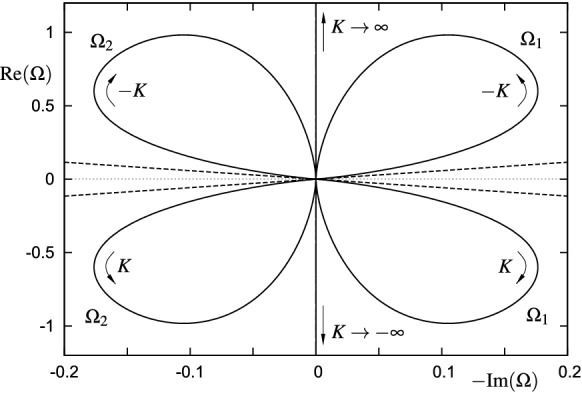
The two roots of the dispersion relations (47) and (62). Solid lines: $\varOmega _1(K)$ (unstable) and $\varOmega _2(K)$ (stable) for the composite model with $C=1/4$; $\text{ Im }(\varOmega )=0$ if $|K|\ge 4$, origin: $|K|=(0,4)$. Dashed lines: asymptotes corresponding to the original relation (47)

On substituting ${\partial {{\mathscr {P}}}_\mathrm{c}}/{\partial x}$ into (45) and rescaling *k* and the angular frequency $\omega$ in a very similar way as in (41) but now with respect to ${\text{ Re }}$ such that $k=\varepsilon ^{-3}K$ and $\omega =\varepsilon ^{-5}\,\varOmega /2$, we obtain the modified and perturbation parameter-free dispersion relation for the compound model

62 $$\begin{aligned} {\varOmega }^{2}+\pi {K}^{2}\sqrt{\varOmega K}+\pi K\varOmega \sqrt{\varOmega K}- C\pi |K|^{3}\sqrt{\varOmega K}=0, \end{aligned}$$

with the last two terms on the left-hand side resulting from the higher order terms in (61). Inspection shows that for $|K|\ge {C}^{-1}$, the modes associated with both roots become neutral, see Fig. 14, and thus the growth rates $-\text{ Im }(\varOmega )$ are bounded from above. This, in turn, gives good reason to assume that the nonlinear Cauchy problem based on this corrected model is well posed. Given the fact that all terms in (62) are of the same order of magnitude, one can furthermore conclude that as soon as the temporal and spatial scales become so small that $x\sim \varepsilon ^3$ and $t\sim \varepsilon ^5$ the problem will be well posed by itself, i.e. without the necessity to include additional regularization terms. This occurs when ${\tilde{x}}/{\tilde{L}}\propto \varepsilon ^4 x\sim O({\text{ Re }}^{-1/2})$ and ${\tilde{t}}\,{\tilde{u}}_\infty /{\tilde{L}}\propto \varepsilon ^2\, t\sim O({\text{ Re }}^{-1/2})$, which are precisely the scalings of the Eulerian stage assumed to follow the present one, see Sect. 5.

### Numerical evidence of regularization

Detailed investigations of the numerical experiments that led to the results presented in Sect. 3.1 indicate that for the spatio-temporal ranges and resolutions used so far, the small but, nonetheless, always present terms resulting from the discretization based on Chebyshev polynomials are very effective in suppressing short-scale instabilities with unbounded growth rates. However, the computation of solutions that blow up in finite time is not feasible with standard methods, due to the required fine resolution of the domains in the neighbourhood of the singularity. In particular, approximations based on polynomial representations are known not to be well suited in this regard. Consequently, the apparently inherent numerical regularization cannot be taken for granted when the solutions approach their assumed finite-time breakdown and grid refinements are necessary. In order to investigate the predicted regularization effect of the modified interaction law (61) according to the composite model derived above, the numerical scheme already developed for the analysis of the two-dimensional triple-deck problem (7) was extended appropriately. Above all, care has to be taken that the damping of small-wavelength disturbances by incorporating higher order terms is not overdone as this could give rise to nonphysical delays in the development of the blow-up solution or nonphysical results at all. The crucial points to be considered in this respect are, first, to quantify the regularization mechanisms already contributed by the numerical scheme. Secondly, then the size of the higher order corrections as given in (61) have to be adjusted in an adaptive way in order for the problem to remain well posed in each time step.

**Fig. 15:**
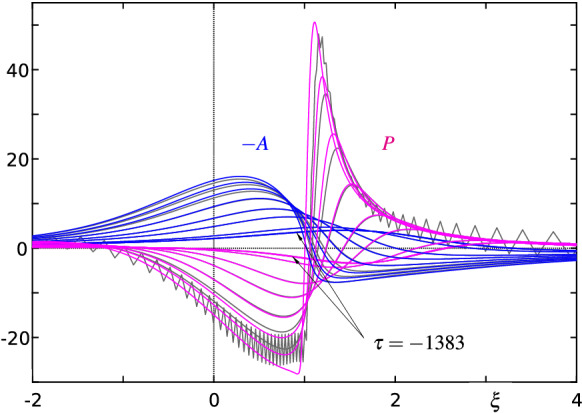
Effect of regularization, (61), with $\varepsilon =0.1$ on the evolution of $A(\xi ,\tau )$ and $P(\xi ,\tau )$ at $\tau =-(1383, 88.5, 22.7, 16.0, 12.2, 10.3, 9.25, 8.50)$. Grey lines display the corresponding results for $\varepsilon =0$ at $\tau =-(1383, 88.5, 22.5, 15.8, 12.0, 10.4, 9.26, 8.46)$. Spatial resolution $m\times n=180\times 90$

**Fig. 16:**
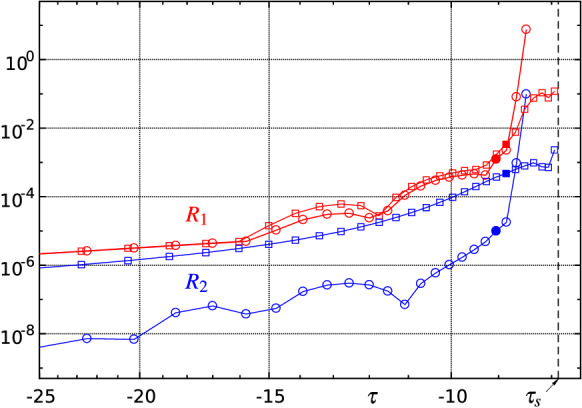
Effect of regularization, (61), on the error indicators $R_1(\tau )$ (red) and $R_2(\tau )$ (blue) according to (32) for a spatial resolution of $m\times n=180\times 90$: $\circ$—$\varepsilon =0$, $\Box$—$\varepsilon =0.1$. Full symbols indicate the upper limit of reliable solutions; predicted blow-up time $\tau _\mathrm{s}$

**Fig. 17:**
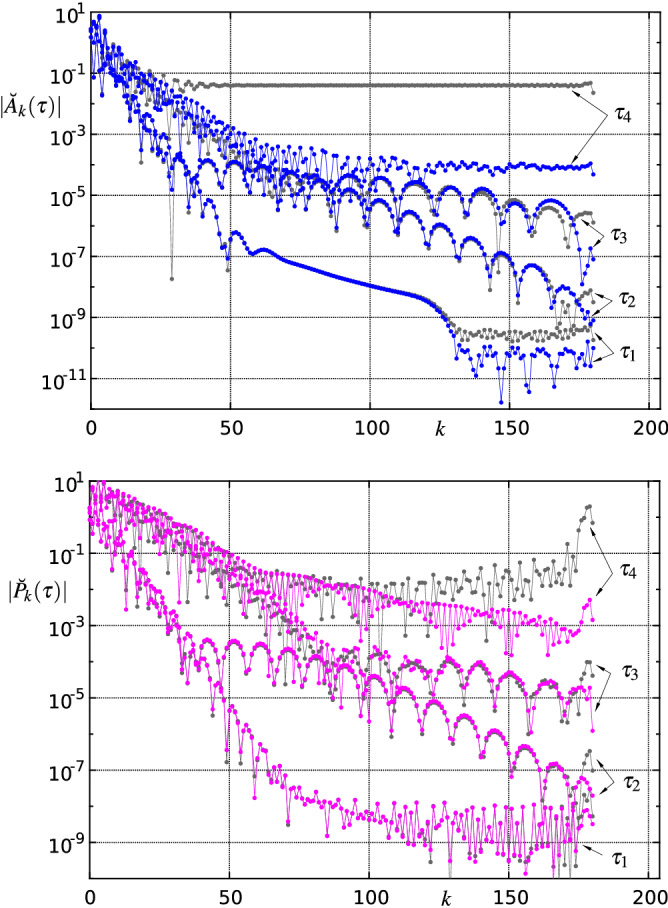
Effect of regularization: modulus of the amplitude spectra (33) of $A(\xi ,\tau )$ and $P(\xi ,\tau )$ for fixed grid resolution $m\times n=180\times 90$ at various time steps. $\varepsilon =0.1$ (coloured), $\tau _l=-(1384, 22.8, 9.25, 8.49)$; $\varepsilon =0$ (grey), $\tau _l=-(1384, 22.5, 9.26, 8.46)$, $l=1,2,3,4$

Numerical experiments with $\varepsilon =0.001$ and $\varepsilon =0.01$ showed practically no differences to the case $\varepsilon =0$ and therefore indicate an (unintentional) intrinsic regularization of the numerical scheme used. An additional filtering is observed when $\varepsilon$ is raised above about 0.01...0.05 (depending on the resolution). A concrete sample calculation demonstrating the effect of regularization is depicted in Figs. 15–17. The results for the time steps shown clearly indicate the effective suppression of grid oscillations, i.e. the expected low-pass filter behaviour of the extended interaction law (61). Since the value selected here for the regularization parameter $\varepsilon =0.1$ is relatively large, there is certainly cause for concern as to whether the solutions in question can still be classified as physically correct. For obvious reasons, $\varepsilon$ should not be larger than absolutely necessary.

## Outlook: Vortex wind up (*Euler–Prandtl stage IV*)

The theory, outlined in Sects. 2 and 3, so far appears to agree with experimental observations regarding the early stages of the laminar–turbulent transition in separation bubbles (cf. Figs. 1 and 2 ), in the sense that it shows how an initially slender, nearly steady, reverse flow eddy of length scale much greater than the boundary layer thickness changes dynamically, through viscous–inviscid interaction mechanisms, into a thick blunt eddy spanning the whole boundary layer in the *noninteracting*, incompressible, fully nonlinear Euler–Prandtl stage formulated below.

Following the lines of reasoning put forward in [29], one can show that, in the triple-deck stage, the evolution of the flow towards the finite-time blow-up as $t\rightarrow t_\mathrm{s}$ is accompanied by a growth of the (originally viscous) sublayer at such a rate that ${\tilde{y}}/{\tilde{L}}\sim {\text{ Re }}^{-4/7}(t_\mathrm{s}-t)^{-1/5}$, until its thickness is indistinguishable from the majority of the boundary layer, i.e. ${\tilde{y}}/{\tilde{L}}\sim {\text{ Re }}^{-1/2}$, cf. Fig. 2. This breakdown implies a new, predominantly inviscid, structure and occurs when the time interval $(t_\mathrm{s}-t)$ reduces to $O({\text{ Re }}^{-5/14})$. With new (local) scalings then given by $({\bar{x}},{\bar{y}},{\bar{t}})\sim {\text{ Re }}^{1/2}({\tilde{x}},{\tilde{y}},{\tilde{t}}{\tilde{u}}_\infty )/{\tilde{L}}$, the relevant velocity components $(u,v)\sim (\psi _{00}^\prime +\psi _{{\bar{y}}},-\psi _{{\bar{x}}})+\cdots$ as well as the pressure $p({\bar{x}},{\bar{y}},{\bar{t}})$ become *O*(1) quantities and are subjected to a fully nonlinear evolution where $u^2\sim v^2\sim p$. The governing equations of this new stage in terms of the stream function $\psi ({\bar{x}},{\bar{y}},{\bar{t}})$ simplify to the Euler equations

63 $$\begin{aligned}&\displaystyle \frac{\partial ^2\psi }{\partial {\bar{y}}\partial {\bar{t}}}+\left( \psi _{00}^\prime +\frac{\partial \psi }{\partial {\bar{y}}}\right) \frac{\partial ^2\psi }{\partial {\bar{y}}\partial {\bar{x}}}-\frac{\partial \psi }{\partial {\bar{x}}}\left( \psi _{00}^{\prime \prime }+\frac{\partial ^2\psi }{\partial {\bar{y}}^2}\right) =-\frac{\partial p}{\partial {\bar{x}}},\nonumber \\&\displaystyle -\frac{\partial ^2\psi }{\partial {\bar{x}}\partial {\bar{t}}}-\left( \psi _{00}^\prime +\frac{\partial \psi }{\partial {\bar{y}}}\right) \frac{\partial ^2\psi }{\partial {\bar{x}}^2}+\frac{\partial \psi }{\partial {\bar{x}}}\frac{\partial ^2\psi }{\partial {\bar{x}}\partial {\bar{y}}}=-\frac{\partial p}{\partial {\bar{y}}} \end{aligned}$$

subject to the boundary conditions $\psi =0$ at ${\bar{y}}=0$, $\psi \rightarrow O(1)$ as ${\bar{y}}\rightarrow \infty$. Here, the classical boundary layer separation profile $\psi _{00}^\prime ({\bar{y}})=U(x_0,{\bar{y}})$ at the point of marginal separation $x_0$ acts as a rotational ‘convective background’ to the inviscid main flow. Again, a viscous sublayer is necessary to bring the slip at the bottom to rest: introducing the appropriate wall-normal coordinate $\eta ={\text{ Re }}^{3/4}{\tilde{y}}/{\tilde{L}}$ results in the spatio-temporal evolution of the stream function $\varPsi ({\bar{x}},\eta ,{\bar{t}})$ and the velocities $u\sim \varPsi _\eta \sim O(1)$, $v\sim -{\text{ Re }}^{-1/4}\varPsi _{{\bar{x}}}$ being determined by Prandtl’s boundary layer equation

64 $$\begin{aligned} \frac{\partial ^2\varPsi }{\partial \eta \partial {\bar{t}}}+\frac{\partial \varPsi }{\partial \eta }\frac{\partial ^2\varPsi }{\partial \eta \partial {\bar{x}}}-\frac{\partial \varPsi }{\partial {\bar{x}}}\frac{\partial ^2\varPsi }{\partial \eta ^2}=-\frac{\partial P}{\partial {\bar{x}}}+\frac{\partial ^3\varPsi }{\partial \eta ^3} \end{aligned}$$

subject to $\varPsi =\varPsi _\eta =0$ at $\eta =0$. Furthermore, $\varPsi \sim B({\bar{x}},{\bar{t}})\eta +\cdots$ as $\eta \rightarrow \infty$, where *B* denotes the slip velocity which is related to the imposed pressure $P({\bar{x}},{\bar{t}})=p({\bar{x}},{\bar{y}}=0,{\bar{t}})$ via Bernoulli’s equation $-P_{{\bar{x}}}=B_{{\bar{t}}}+BB_{{\bar{x}}}$. The initial (matching) conditions of (63), (64) as ${\bar{t}}\rightarrow -\infty$ are fixed by

65 $$\begin{aligned} \psi \sim |{\bar{t}}|^{-3/5}\left[ {\hat{\psi }}({\hat{x}},{\hat{y}})+\cdots \right] ,\quad p\sim |{\bar{t}}|^{-4/5}\left[ \hat{{\mathscr {P}}}({\hat{x}})+\cdots \right] ,\quad \varPsi \sim |{\bar{t}}|^{1/10}\left[ {\hat{\varPsi }}({\hat{x}},{\hat{\eta }})+\cdots \right] \end{aligned}$$

and the similarity variables ${\bar{x}}=|{\bar{t}}|^{3/5}{\hat{x}}$, ${\bar{y}}=|{\bar{t}}|^{-1/5}{\hat{y}}$, and $\eta =|{\bar{t}}|^{1/2}{\hat{\eta }}$. Here, the leading order terms are determined by the triple-deck blow-up profiles ${\hat{\psi }}({\hat{x}},{\hat{y}})$, $\hat{{\mathscr {P}}}({\hat{x}})$, and ${\hat{\varPsi }}({\hat{x}},{\hat{\eta }})$, which satisfy (37) and (39), respectively, see Figs. 12 and 13 . More precisely, the explicit quotation of $\psi _{00}$ in (63) leads to a slightly modified formulation of the blow-up profiles, but without loss of generality.

Current efforts aim, at first, to compute (at least) the next-order corrections in (65) in order for the associated initial value problems to be defined properly, and, secondly, to construct a suitable numerical scheme able to capture the vortex wind-up process governed by (63) and (64). As in the triple-deck stage, the infinite spatial and temporal domains and the initial similarity structure require special treatment, cf. (21). According to the local expansions of the boundary layer characteristics (2) in the Euler–Prandtl stage

66 $$\begin{aligned} \delta ^*\sim \delta ^*(x_0)-U_{00}^{-1}\lim _{{\bar{y}}\rightarrow \infty }\psi +\cdots ,\quad \tau _\mathrm{w}\sim {\text{ Re }}^{1/4}\varPsi _{\eta \eta }\Big |_{\eta =0}+\cdots \end{aligned}$$

we expect *O*(1) corrections to the original displacement thickness and a substantially enhanced action of the wall shear stress.

Although we started the investigation of transitional separation bubbles with the analysis of formally *steady* classical boundary layer flows on the verge of separation, rather weak unsteady forcing (blowing) in the first, large-scale, interaction stage of marginal separation eventually led us (via a second, shorter-scale triple-deck interaction) to a scenario that in literature is commonly referred to as *unsteady separation*, see the reviews [79, 80] and references therein. In sharp contrast to the usual setting underlying the model problems studied in the context of unsteady separation, the outer inviscid flow field is *not* given in advance in the current formulation (63), (64). The spatio-temporal evolution of the flow is entirely *self-induced* once kicked by the initial condition (65). Nevertheless, from solving the Cauchy problems (63)–(65) we expect the occurrence of the generic behaviour known from the investigation of unsteady boundary layer flows with prescribed pressure gradient: the abrupt eruption of near-wall fluid (vorticity) out of the viscous boundary layer in form of a finite-time blow-up. The existence of this singular behaviour was first reported by Blasius [81] in his study of an impulsively started circular cylinder. Another example was given by Walker [82] who studied the finite-time singularity in the boundary layer exposed to a rectilinear vortex. Then these results were re-examined numerically and analytically by various authors, including [83–89] and [90].

As pointed out in [79], however, a Rayleigh-type instability may arise in the Euler region well before a singularity develops in the noninteractive boundary layer evolution (64), see also [91]. From a computational point of view, a Lagrangian-type scheme might be advantageous compared to the more conventional Eulerian formulations [87, 92], although more recently the latter approach also proved suitable [93].

## Concluding remarks

The present paper essentially is a revision of the fundamental work of Elliott and Smith [29]. A spectral collocation method based on Chebyshev polynomials has been developed for treating the second—shorter—interactive (triple deck) stage of marginally separated flows. Special emphasis was placed on solutions that blow up within finite time. The predicted blow-up structure could be confirmed with a high level of evidence. To this end, the high wave number behaviour of the amplitude spectrum of relevant quantities proved to be particularly suitable for assessing the reliability of the numerical solutions. The numerical scheme implemented turned out to regularize the proven ill-posedness of the underlying Cauchy problem, i.e. the tendency of solutions to unbounded growth of short-scale disturbances is sufficiently suppressed. Additionally, we have shown that a suitable modification of the usual interaction law by means of a so-called composite asymptotic model again contributes to a successful suppression of the predicted short-scale instability.

Although the asymptotic theory of marginal separation and its extension to a triple-deck stage is established for more than three decades, we believe it is a key approach able to unveil the secrets of the laminar–turbulent transition process in separation bubbles. Still, its potential is by far not exploited and important issues still remain open, which keeps this research area in a state of excitement. From our current perspective, these points comprise the following: *(i)* Treatment of ill-posed Cauchy problems associated with the asymptotic theory of separated flows, *(ii)* tracing the breakdown cascade further: vortex wind-up and shedding in the Euler–Prandtl stage, *(iii)* Providing tools for transition detection and control.

In order to keep track of the big picture and, at the same time, to shed light on the achievements of the asymptotic theory, we inspect the ${\text{ Re }}$-dependence of typical quantities characterizing the status of the boundary layer with respect to laminar–turbulent transition. Such an approach may also serve as source of suggestions and reference points for laboratory experiments and computational fluid dynamics. For example, the theory of marginal separation (stage II) predicts a separation bubble length of $O({\text{ Re }}^{-1/5})$, an initial (‘steady’) bubble height of $O({\text{ Re }}^{-7/10})$ and the slope of the (almost) steady separation streamline to be of $O({\text{ Re }}^{-1/2})$, which are facts that still do not seem to be commonly known, cf. the comments in [94]. From the triple-deck model (stage III), a length of $O({\text{ Re }}^{-2/7})$ and a height of $O({\text{ Re }}^{-4/7})$ can be estimated for the emerging spike at the rear of the bubble, which is consequently still characterized as a slender phenomenon. In the Euler–Prandtl stage IV, both the length and height of the shedding vortex then reach $O({\text{ Re }}^{-1/2})$, comparable to the thickness of the original boundary layer. Table 2 illustrates the successive order-of-magnitude reductions of the relevant time scales, the streamwise length scales, the viscous wall layer thickness and the enhancement of the wall friction velocity $u_\tau =[{\tilde{\tau }}_\mathrm{w}/({\tilde{\rho }}{\tilde{u}}_\infty ^2)]^{1/2}$ during incipient transition. Our admittedly ambitious long-term objective is to fully bridge the gap between the established concepts of both the laminar boundary layer and the time-averaged (fully developed, attached) two-tiered turbulent boundary layer, [30, 31] (and the references therein), via a correct asymptotic description of the whole transition process.

**Table 2:** Order of magnitude alterations of selected quantities during the early stages of bypass transition triggered by incipient separation in the limit as ${\text{ Re }}\rightarrow \infty$ ($x\rightarrow x_0$, $\alpha \rightarrow \alpha _\mathrm{c}$), cf. Fig. 2

Stage	I	II	III	IV	Final$^{\dagger }$
Time scale, ${\tilde{t}}{\tilde{u}}_\infty /{\tilde{L}}$	–	${\text{ Re }}^{+1/20}$	${\text{ Re }}^{-1/7}$	${\text{ Re }}^{-1/2}$	–
Length scale, ${\tilde{x}}/{\tilde{L}}$	${\text{ Re }}^0$	${\text{ Re }}^{-1/5}$	${\text{ Re }}^{-2/7}$	${\text{ Re }}^{-1/2}$	${\text{ Re }}^0$
Viscous layer thickness, ${\tilde{y}}/{\tilde{L}}$	${\text{ Re }}^{-1/2}$	${\text{ Re }}^{-11/20}$	${\text{ Re }}^{-4/7}$	${\text{ Re }}^{-3/4}$	${\text{ Re }}^{-1}\ln {\text{ Re }}$
Pressure, $({\tilde{p}}-{\tilde{p}}_\infty )/({\tilde{\rho }}{\tilde{u}}_\infty ^2)\quad$	${\text{ Re }}^0$	${\text{ Re }}^{-1/2}$	${\text{ Re }}^{-2/7}$	${\text{ Re }}^0$	${\text{ Re }}^0$
Wall friction velocity, $u_\tau$	${\text{ Re }}^{-1/4}$	${\text{ Re }}^{-7/20}$	${\text{ Re }}^{-2/7}$	${\text{ Re }}^{-1/8}$	$\ln ^{-1}{\text{ Re }}$

I—steady classical boundary layer, II—marginal separation, III—triple deck stage (spike formation), IV—Euler–Prandtl stage (vortex wind-up), $^{\dagger }$Time-averaged, fully developed wall-bounded turbulence, see e.g. [30]

### Supplementary Information

Below is the link to the electronic supplementary material.

Supplementary material 1 (pdf 99 KB)

Supplementary material 2 (dat 88 KB)

Supplementary material 3 (avi 22117 KB)

## Figures and Tables

**Fig. 18:**
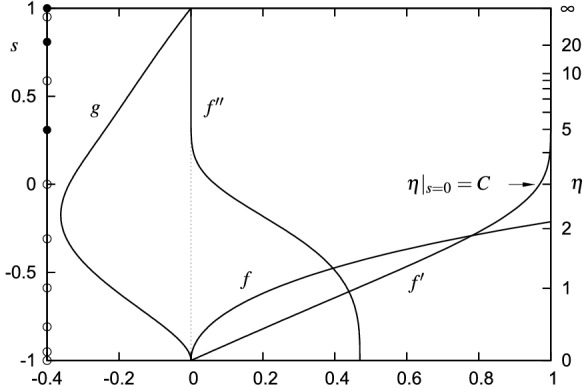
Flat plate laminar boundary layer (Blasius flow) test case: solutions for the bounded auxiliary function *g*, stream function *f*, velocity profile $f^\prime$ and shear stress (vorticity) $f^{\prime \prime }$. Gauss–Lobatto Chebyshev nodes $s_j$, here demonstrated for $n=10$ (left ordinate): open and full circles denote the collocation points where equation (76) and the boundary conditions $g(0)=g'(0)=0$, as well as (77) are evaluated, respectively. Chosen mapping parameters: $C=3$, $\eta ^*=0$, see (16)

## Appendix A: A brief formulation of the triple-deck stage

The following description supplement the pivotal work of [29] with asymptotic expansions for the different spatial regions of the triple-deck stage (see Fig. 2) and affine transformations. Dimensionless quantities are introduced in the form

67 $$\begin{aligned} (x,y)=\frac{({\tilde{x}},{\tilde{y}})}{{\tilde{L}}},\quad t=\frac{{\tilde{u}}_\infty {\tilde{t}}}{{\tilde{L}}},\quad (u,v)=\left( \frac{\partial \psi }{\partial y},-\frac{\partial \psi }{\partial x}\right) =\frac{({\tilde{u}},{\tilde{v}})}{{\tilde{u}}_\infty }, \quad p=\frac{{\tilde{p}}-{\tilde{p}}_\infty }{{\tilde{\rho }}{\tilde{u}}_\infty ^2}, \end{aligned}$$

where *x*, *y*, and *u*(*x*, *y*, *t*), *v*(*x*, *y*, *t*) denote the coordinates and velocity components in the streamwise direction and normal to the wall, *t* the time, $\psi (x,y,t)$ the stream function and *p*(*x*, *y*, *t*) the pressure, respectively. The unperturbed free stream values ${\tilde{u}}_\infty$, ${\tilde{p}}_\infty$, density ${\tilde{\rho }}$ and a characteristic length ${\tilde{L}}$ are used as (dimensional) reference quantities. The corresponding spatio-temporal scalings and expansions of the relevant field quantities were read as follows.

### A.1 Lower deck LD

In this viscous sublayer, the crucial dynamics of the spike formation process takes place after being initiated through a blow-up event in the preceding marginal separation stage. Local coordinates in streamwise and wall-normal direction and the time are given by

68 $$\begin{aligned} {\check{x}}={\text{ Re }}^{2/7}\left( x-x_0-{\text{ Re }}^{-1/5}X_\mathrm{s}\right) ,\quad {\check{y}}={\text{ Re }}^{4/7}y,\quad {\check{t}}={\text{ Re }}^{1/7}\left( t-{\text{ Re }}^{1/20}T_\mathrm{s}\right) . \end{aligned}$$

Asymptotic expansions for the stream function and the pressure read

69 $$\begin{aligned} \begin{array}{l} \psi \sim {\text{ Re }}^{-5/7}{{\check{\psi }}}({\check{x}},{\check{y}},{\check{t}})+\cdots ,\\ p\sim (1-U_{00}^2)/2+p_{00}\left( {\text{ Re }}^{-1/5}X_\mathrm{s}+{\text{ Re }}^{-2/7}{\check{x}}\right) +\cdots +{\text{ Re }}^{-2/7}\check{{\mathscr {P}}}({\check{x}},{\check{t}})+\cdots . \end{array} \end{aligned}$$

Here $x_0$ denotes the (marginal) separation point according to classical boundary layer theory in the limit as the control parameter $\alpha$ attains its critical value $\alpha _\mathrm{c}$ and $(X_\mathrm{s},T_\mathrm{s})$ the blow-up location/time of the marginal separation stage II. Furthermore, $U_{00}=u_\mathrm{w}(x_0)>0$ represents the wall velocity of the steady outer inviscid flow and $p_{00}>0$ the imposed adverse pressure gradient at $x_0$ which enter the Taylor series expansion of the Bernoulli equation $p_\mathrm{w}(x)=[1-u_\mathrm{w}^2(x)]/2$ for the wall streamline in the limit as $x-x_0\rightarrow 0$.

### A.2 Main deck MD

This predominantly inviscid, rotational region passively shifts the displacement effect generated in the lower deck to the upper deck. Likewise, the pressure response induced in the upper deck is transmitted back to the lower deck via this layer. The relevant wall normal coordinate is the usual Prandtl boundary layer coordinate

$$\begin{aligned} {\bar{y}}={\text{ Re }}^{1/2}y, \end{aligned}$$

and the corresponding expansions are

70 $$\begin{aligned} \begin{array}{l} \psi \sim {\text{ Re }}^{-1/2}\left[ \psi _{00}({\bar{y}})+\psi _{01}({\bar{y}})\left( {\text{ Re }}^{-1/5}X_\mathrm{s}+{\text{ Re }}^{-2/7}{\check{x}}\right) +\cdots \right] +{\text{ Re }}^{-4/7}\bar{{\mathscr {A}}}({\check{x}},{\check{t}})\psi _{00}^\prime +\cdots ,\\ p\sim (1-U_{00}^2)/2+p_{00}\left( {\text{ Re }}^{-1/5}X_\mathrm{s}+{\text{ Re }}^{-2/7}{\check{x}}\right) +\cdots +{\text{ Re }}^{-2/7}\bar{{\mathscr {P}}}({\check{x}},{\check{t}})+\cdots . \end{array} \end{aligned}$$

Here $\psi _{00}^\prime ({\bar{y}})$ and $\psi _{01}^\prime ({\bar{y}})$ denote the boundary layer separation (velocity) profile and the first Taylor term (see e.g. [42], p. 149 et seq., and p. 160) with the properties $\psi _{00}^\prime \rightarrow U_{00}$, $\psi _{01}^\prime \rightarrow -p_{00}/U_{00}$ as ${\bar{y}}\rightarrow \infty$.

### A.3 Upper deck UD

The streamwise (${\check{x}}$) and wall-normal coordinate

$$\begin{aligned} {\hat{y}}={\text{ Re }}^{2/7}y \end{aligned}$$

are equally scaled in this potential flow region with the concerning expansions

71 $$\begin{aligned} \begin{array}{l} u\sim \displaystyle U_{00}-\frac{p_{00}}{U_{00}}\left( {\text{ Re }}^{-1/5}X_\mathrm{s}+{\text{ Re }}^{-2/7}{\check{x}}\right) +\cdots +{\text{ Re }}^{-2/7}{\hat{u}}({\check{x}},{\hat{y}},{\check{t}})+\cdots ,\\ v\sim \displaystyle {\text{ Re }}^{-2/7}\frac{p_{00}}{U_{00}}{\hat{y}}+\cdots +{\text{ Re }}^{-2/7}{\hat{v}}({\check{x}},{\hat{y}},{\check{t}})+\cdots ,\\ p\sim (1-U_{00}^2)/2+p_{00}\left( {\text{ Re }}^{-1/5}X_\mathrm{s}+{\text{ Re }}^{-2/7}{\check{x}}\right) +\cdots +{\text{ Re }}^{-2/7}\hat{p}({\check{x}},{\hat{y}},{\check{t}})+\cdots \end{array} \end{aligned}$$

containing the perturbation velocity components ${\hat{u}},{\hat{v}}$ and the induced pressure $\hat{p}$ ($={\hat{p}}_1$ of Sect. 4).

### A.4 Affine transformations and fundamental triple-deck formulation

From the individual substitution of (69)–(71) into the Navier–Stokes equations and the application of the matching principle one obtains ${\hat{p}}({\check{x}},{\hat{y}}=0,{\check{t}})=\bar{{\mathscr {P}}}({\check{x}},{\check{t}})=\check{{\mathscr {P}}}({\check{x}},{\check{t}})$ and, on further using the affine transformations

72 $$\begin{aligned} \begin{array}{c} {\check{x}}\rightarrow p_{00}^{-5/7}U_{00}^{8/7}x,\quad {\check{y}}\rightarrow p_{00}^{-3/7}U_{00}^{2/7}y,\quad {\check{t}}\rightarrow p_{00}^{-6/7}U_{00}^{4/7}t,\\ {{\check{\psi }}}\rightarrow p_{00}^{-2/7}U_{00}^{6/7}\psi ,\quad \bar{{\mathscr {P}}}\rightarrow p_{00}^{2/7}U_{00}^{8/7}{{\mathscr {P}}},\quad \bar{{\mathscr {A}}}\rightarrow p_{00}^{4/7}U_{00}^{2/7}{{\mathscr {A}}}, \end{array} \end{aligned}$$

the lower-deck equation (7) and the interaction law (8) from the upper deck problem.

## Appendix B: Chebyshev collocation method for unbounded domains: Blasius flow test problem

The well-known and rather simple flat plate (Blasius) similarity boundary layer flow problem is used as an application example to demonstrate the power and essence of the Chebyshev collocation method representing the heart of Sect. 3. At first, the semi-infinite physical domain (in wall-normal direction) is mapped onto the finite computational (Chebyshev) domain. Furthermore, the sought stream function is split into two parts such that the intrinsic unknown, to be represented by Chebyshev polynomials, is strictly bounded.

In the following we want to solve the nonlinear two-point boundary value problem

73 $$\begin{aligned} f^{\prime \prime \prime }+ff^{\prime \prime }=0,\quad f(0)=f'(0)=0,\quad f'(\eta \rightarrow \infty )\rightarrow 1, \end{aligned}$$

where the usual boundary layer stream function $\psi (x,{\bar{y}})=\sqrt{2x}\,f(\eta )$ is reduced to the similarity form $f(\eta )$ with the similarity variable $\eta ={\bar{y}}/\sqrt{2x}$. As is well known, see e.g. [95], its far-field behaviour is given by

74 $$\begin{aligned} f(\eta \rightarrow \infty )\sim \zeta +O(\text {e}^{-\zeta ^2/2}/\zeta ^2), \end{aligned}$$

where we have used the abbreviation $\zeta =\eta -\beta _1$. The value of the constant $\beta _1=\lim _{\eta \rightarrow \infty }(\eta -f)$ to be determined in the course of the numerical solution procedure, fixes the displacement thickness (2) to $\delta ^*(x)=\sqrt{2x}\,\beta _1$. In order to capture the singular behaviour of *f* as $\eta \rightarrow \infty$, we therefore may write

75 $$\begin{aligned} f(\eta )=g(\eta )+\frac{\eta ^2}{\beta _1+\eta } \end{aligned}$$

to obtain

76 $$\begin{aligned} g^{\prime \prime \prime }-\frac{6\beta _1^2}{(\beta _1+\eta )^4}+\left( \frac{\eta ^2}{\beta _1+\eta }+g\right) \left( \frac{2\beta _1^2}{(\beta _1+\eta )^3}+g^{\prime \prime }\right) =0 \end{aligned}$$

for the bounded auxiliary function *g*: $g(0)=g^\prime (0)=g(\infty )=0$. Taking into account the mapping (16) for the wall-normal direction, $\eta =\eta (s)$ with no shift $\eta ^*=0$, we equate $\mathrm{d}g/\mathrm{d}s=(\mathrm{d}g/\mathrm{d}\eta )(\mathrm{d}\eta /\mathrm{d}s)$ using $\mathrm{d}\eta /\mathrm{d}s=\pi (\eta ^2/C+C)/4$ and $\mathrm{d}g/\mathrm{d}\eta \sim \beta _1^2/\eta ^2+O(\eta ^{-3})$ as $\eta \rightarrow \infty$ to deduce the exact result

77 $$\begin{aligned} \frac{\mathrm{d}g}{\mathrm{d}s}\bigg |_{s=1}=\frac{\beta _1^2\pi }{4C}. \end{aligned}$$

To compute the $(n+1)$ unknowns ($g_0,g_1,\ldots ,g_{n-1},\beta _1$), at the collocation points $s_i$, (76) is discretized with the help of the differentiation matrices (17) for the $\eta$-domain and (77) is implemented via (14),

78 $$\begin{aligned} \sum _{j=1}^{n-1}D_{nj}^{(1)}g_j - \frac{\beta _1^2\pi }{4C} = 0. \end{aligned}$$

The whole nonlinear algebraic system of equations then is solved by means of the NAG c05qbc routine. The corresponding results are plotted in Fig. 18.

To check the accuracy/reliability of the obtained results, it is useful to monitor the residuals of the discretized equations (76) omitted at the two interior locations which are used to implement the boundary conditions $g(0)=g^\prime (0)=0$ (represented by the full circles in Fig. 18). Furthermore, alternative accuracy and consistency checks on $\beta _1$ may be performed via the relations

79 $$\begin{aligned} \int _0^\infty g'\,\mathrm{d}\eta =0,\quad g^{\prime \prime \prime }(0)=\frac{6}{\beta _1^2}. \end{aligned}$$

Here the integration is preferably carried out with the Clenshaw–Curtis quadrature, whose weights can be computed analytically, see e.g. [67]. In our experience, the technique described above already gives 4-digit accuracy for $n\approx 20$, impressively demonstrating the superiority of Chebyshev spectral methods, table 3.

**Table 3:** Blasius flow characteristics displacement thickness $\delta ^*(x)=\sqrt{2x}\beta _1$ and wall shear stress $\tau _\mathrm{w}(x)=f^{\prime \prime }(0)/\sqrt{2x}$ in dependence of the number of Chebyshev collocation points *n* with $C=3$

*n*	10	20	40	80	Reference value [96]
$\beta _1$	1.2	1.2165	1.216776	1.216780622	1.216780621614862
$f^{\prime \prime }(0)$	0.52	0.46957	0.4696001	0.4695999884	0.4695999883610133

## Footnotes

1 During preparation of this manuscript, Servini, at that time PhD student at the University College London, kindly drew the authors’ attention to an erroneous appearance of the obstacle shape function *h* in the fundamental equation presented in [43], which was corrected accordingly in (3).

2 Chebfun—open-source package for numerical computing with functions (Matlab based), University of Oxford, http://www.chebfun.org/.

3 The NAG C Library, The Numerical Algorithms Group (NAG), Oxford, United Kingdom http://www.nag.com (Mark 26).
