# Supplementary material for: The triple-deck stage of marginal separation
Source: J Eng Math. 2021 Jun 1;128(1):16. doi: 10.1007/s10665-021-10125-3 (PMC8550611; doi:10.1007/s10665-021-10125-3)
Supplement: Supplementary file 1 — Supplementary material 1 (pdf 99 KB) [file 10665_2021_10125_MOESM1_ESM.pdf]

Supplementary material

Please note that supplementary electronic material has been added to this manuscript:
[1] Airfoil coordinates data (M.Trenker_airfoil.dat) used to generate the results plotted in figure 3.
[2] A video file (psi.avi) showing the development of the contour lines of the stream function over time for the triple-deck stage.
Description: Illustration of the temporal evolution of the isolines of $\psi(x,y,t)$ according to (7)-(12), computed with a spatial resolution of $m \times n = 230 \times 115$. Separation streamline $\psi = 0$ (bold solid black line), contour lines $\psi = -(0.05, 0.1, 0.2, 0.5, 1, 2, 3, 5)$ (emerging thin solid black lines within the 'spike'), $\psi = 0.1, 1, 10, 100$, cf. figure 9; the trace of the vortex center $\times$ (minimum of ψ) is marked as a thin solid black line. The numerical solution fails beyond $t \gtrsim -8.83$ (5 frames, dotted isolines), indicated by the sharp right-turning of the vortex center.
